# Photo‐responsive Helical Motion by Light‐Driven Molecular Motors in a Liquid‐Crystal Network

**DOI:** 10.1002/anie.202016254

**Published:** 2021-03-12

**Authors:** Jiaxin Hou, Anirban Mondal, Guiying Long, Laurens de Haan, Wei Zhao, Guofu Zhou, Danqing Liu, Dirk J. Broer, Jiawen Chen, Ben L. Feringa

**Affiliations:** ^1^ SCNU-UG International Joint Laboratory of Molecular Science and Displays National Center for International Research on Green Optoelectronics South China Normal University Guangzhou 510006 China; ^2^ Stratingh Institute for Chemistry University of Groningen Nijenborgh 4 9747 AG Groningen The Netherlands; ^3^ SCNU-TUE Joint lab of Device Integrated Responsive Materials (DIRM) Guangdong Provincial Key Laboratory of Optical Information Materials and Technology & Institute of Electronic Paper Displays South China Academy of Advanced Optoelectronics South China Normal University Guangzhou 510006 China; ^4^ Stimuli-responsive Functional Materials and Devices Department of Chemical Engineering and Chemistry Eindhoven University of Technology Den Dolech 2, 5600 MB Eindhoven The Netherlands

**Keywords:** chirality, helical motion, liquid-crystal network, molecular motors, photo-responsive motion

## Abstract

Controlling sophisticated motion by molecular motors is a major goal on the road to future actuators and soft robotics. Taking inspiration from biological motility and mechanical functions common to artificial machines, responsive small molecules have been used to achieve macroscopic effects, however, translating molecular movement along length scales to precisely defined linear, twisting and rotary motions remain particularly challenging. Here, we present the design, synthesis and functioning of liquid‐crystal network (LCN) materials with intrinsic rotary motors that allow the conversion of light energy into reversible helical motion. In this responsive system the photochemical‐driven molecular motor has a dual function operating both as chiral dopant and unidirectional rotor amplifying molecular motion into a controlled and reversible left‐ or right‐handed macroscopic twisting movement. By exploiting the dynamic chirality, directionality of motion and shape change of a single motor embedded in an LC‐network, complex mechanical motions including bending, walking and helical motion, in soft polymer materials are achieved which offers fascinating opportunities toward inherently photo‐responsive materials.

## Graphical abstract

By exploiting the dynamic chirality, directionality of motion and shape change of a single light‐driven motor embedded in a liquid‐crystal network, complex mechanical motions including bending, walking and helical motion, in soft polymer materials are achieved which offers fascinating opportunities toward inherently photo‐responsive materials.

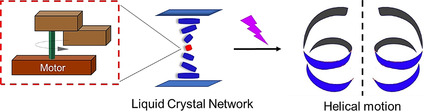

## Introduction

The prospects of dynamic molecular systems,[^1^, ^2^, ^3^] using artificial molecular machines[^4^, ^5^, ^6^] to induce motion and perform complex mechanical tasks reminiscent of the omnipresent “machinery of life”,[^7^, ^8^] has greatly stimulated scientists to design molecular motors and machines that can power specific movements or allow several distinct mechanical operations.[^9^, ^10^] Molecular muscles,[^11^, ^12^, ^13^] chemical synthesizers,[^14^, ^15^] multitasking catalysts,[^16^, ^17^] self‐sorting machines,^[18]^ transporters,[^19^, ^20^] pumps[^21^, ^22^, ^23^] and responsive surfaces[^24^, ^25^] are illustrative of machine‐like dynamic functions demonstrated in recent years. Controlled mechanical movement has been achieved in solution, on surfaces and in materials including rotation,[^26^, ^27^, ^28^] translation[^29^, ^30^, ^31^] and bending[^32^, ^33^] motions. Despite these advances the development of systems in which molecular movement is translated and amplified along multiple length scales to induce macroscopic motion as a basis for actuator materials has been limited.[^34^, ^35^] Facing the challenge to achieve multiple distinctive autonomous motions in a soft material powered by light we envisioned that our photochemical rotary motors[^36^, ^37^] offer unique opportunities to achieve more complex mechanical movements. In particular, these systems allow control of directionality of rotary movement and are driven non‐invasively using light energy.[^36^, ^37^] In addition, designing single molecular motor systems and organizing these in such a way that they can induce various types of motion, reminiscent of distinct mechanical actions by a biological muscle, represents a major next step in the field of molecular machines. Photochromic switches have been used, for example, in responsive crystals,[^38^, ^39^, ^40^, ^41^] polymers,^[42]^ LC materials[^43^, ^44^, ^45^, ^46^, ^47^, ^48^, ^49^, ^50^] and gels,[^51^, ^52^, ^53^] while motors have shown photochemical rotary motion of micro‐objects on LC‐surfaces[^54^, ^55^] and heat‐driven anisotropic deformations of LC films.^[56]^ Katsonis and co‐workers[^57^, ^58^] elegantly demonstrated macroscopic helical tendril‐like motion using an intricate combination of light of distinct wavelengths, an LC‐polymer network, chiral dopant and azobenzene photoswitch taking advantage of specific morphology and alignment in the LC material. In our approach all the key parameters, that is, dynamic chirality, dopant function, shape change and photo‐responsiveness, are embedded in a single rotary motor that acts as a cross‐linker unit in a polymer LC network. Furthermore, a single wavelength of light enables fast and reversible amplification of motion resulting not only in bending and walking but also inducing left‐ or right‐ handed macroscopic twisting motions of the polymer. The precise control of the structure with light and the inherent and dynamic helical chirality (racemic, P or M) of the molecular motor govern the multiple complex movements at the macroscopic level in the material.

## Results and Discussion

### Design and Synthesis

**Figure 1:**
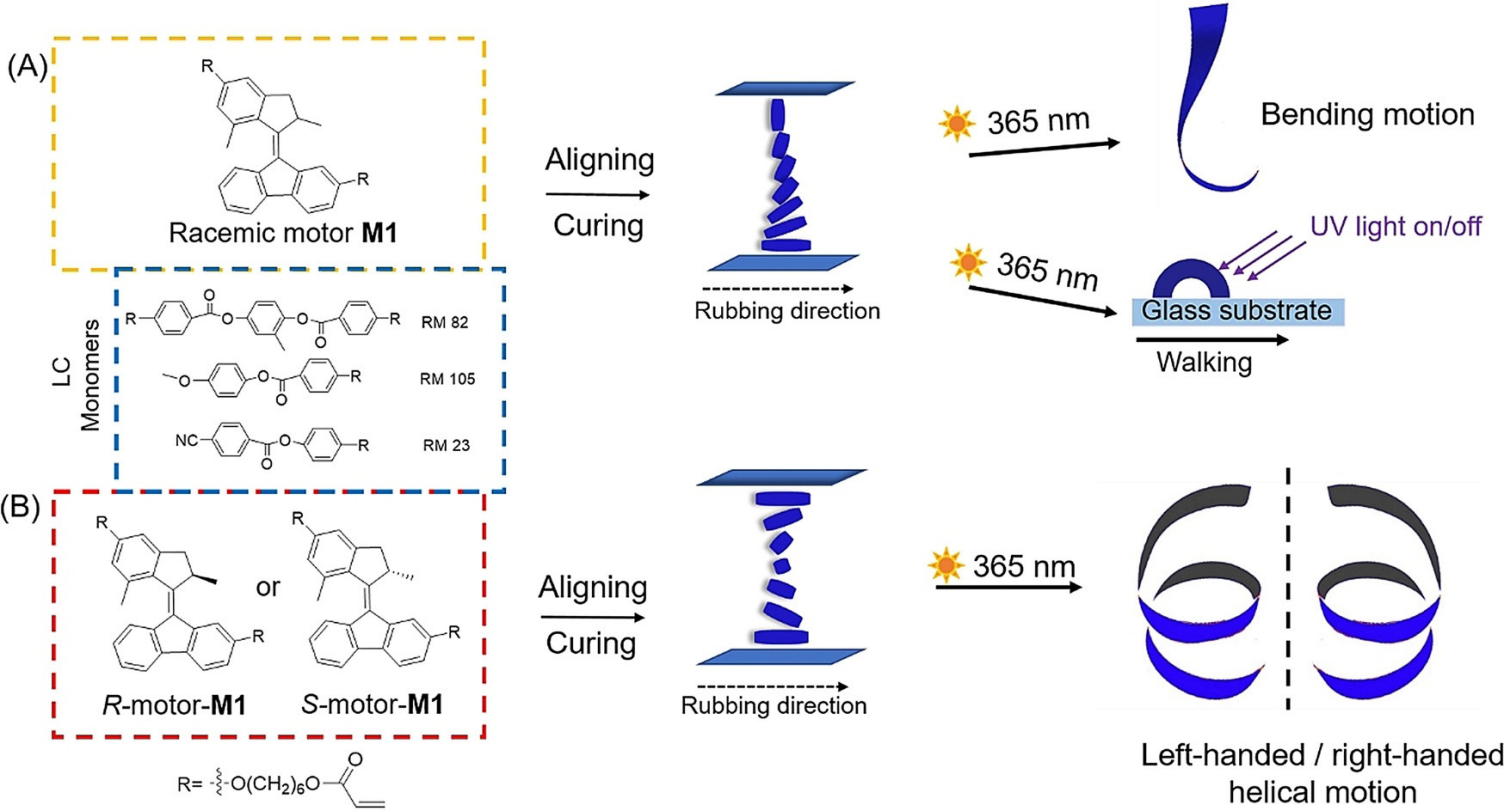
Molecular rotary motor‐based photo‐responsive liquid‐crystal network (LCN) and structure of the LC monomers and molecular motors as crosslinkers. A) LC monomers (RM 82, RM 105, RM 23) (blue dashed square) with racemic motor (*R*,*S*)‐**M1** (yellow dashed square). A mixture of the LC monomers and racemic motor (*R*,*S*)‐**M1** (3 wt %) is aligned from homeotropic to planar. The mixture is cured into a homogeneous film and is cut along the rubbing direction. The obtained ribbon is able to bend upon UV light irradiation (365 nm) or walk over a surface. B) LC monomers with enantiomerically pure motors (*R*)‐**M1** and (*S*)‐**M1** (red dashed square). The LC monomers are mixed with (*R*)‐**M1** or (*S*)‐**M1** motor (1 wt %) and aligned into a twisted nematic structure. The mixture is cured into a homogeneous film and is cut along the rubbing direction. The resulting ribbon with *R*‐motor shows left‐handed helical motion when irradiated with UV light, while the ribbon with *S*‐motor shows right‐handed helical motion.

A liquid‐crystal polymer network forms the basis for our material that reorganizes into macroscopic helices in a fully reversible manner upon illumination (Figure 1). In our approach a molecular rotary motor with distinct upper and lower halves (denoted as a second‐generation motor) **M1** is used as a light‐responsive chiral cross‐linker in an aligned nematic liquid‐crystal polymer network. Key to the functioning of these systems is the photochemical‐induced change of mesoscopic order as the motor **M1** simultaneously functions as chiral LC dopant, controlling the initial helicoidal molecular organization, and the light‐responsive unit inducing major changes in shape and chirality of the polymer. Large deformations are expected as there is a strong coupling between mechanical strain, chirality of the dopant and orientational order in the LC polymer network.

The material used for the LC matrix is obtained by photopolymerization of a mixture of acrylate‐ functionalized nematic liquid crystals (RM 82, RM 105, RM 23)(Figure 1), in a ratio that allows processing of the monomers at moderate temperatures and enough flexibility after polymerization to permit deformation of the polymer films. The designed motor cross‐linker molecule **M1** (Figure 1) contains two essential parts: i) an overcrowded‐alkene‐based second‐generation light‐driven rotary motor as a central core and ii) two acrylate moieties for co‐polymerization in the liquid‐crystal network (Figure 1). In the present study, we employed a second‐generation motor with a cyclopentene upper‐ and a fluorenene‐lower half (Figure 1) as the core structure because motors of related structures have high rotary speeds (*t*_1/2_=1–3 min at rt),^[59]^ which are suitable for fast actuation[^45^, ^53^] and these structures also allow for easy functionalization at both upper and lower halves. A C‐6 carbon spacer was placed between the motor core and the acrylate moieties to act as a bifunctional cross‐linker while providing enough free space for the motor to rotate inside the polymer network (Figure 1). Motor **M2** with a single acrylate functional group was prepared to serve as a control compound (Scheme S1).

**Figure 2:**
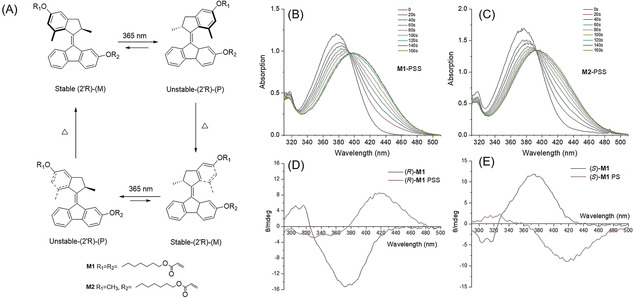
Light‐driven rotation of molecular motor **M1**. A) Rotary cycle of **M1** (only one enantiomer is shown here). Step 1,3: Photoisomerization; step 2,4: Thermal isomerization. B) UV/Vis spectra of **M1** in DCM at 263 K upon UV irradiation (5×10^−5^ M). C) UV/Vis spectra of **M2** in DCM at 263 K upon UV irradiation (5×10^−5^ M). D) CD spectra of (*R*)‐**M1** in DCM at 253 K upon UV irradiation (3.8×10^−5^ M). E) CD spectra of (*S*)‐**M1** in DCM at 253 K upon UV irradiation (3.8×10^−5^ M).

The synthesis of bisacrylate functionalized molecular motor **M1** started with tetralone **3** which was converted in three steps (deprotection, reprotection, thionation) in TBS‐ protected thioketone **6** (Scheme S1). The lower half, ketone **7** was treated with hydrazine monohydrate and the resulting hydrazone **8** oxidized with MnO_2_ at 0 °C to generate the corresponding diazo compound **9**. The key step in the synthesis of the overcrowded alkene is a Barton–Kellogg coupling reaction which requires heating of the thioketone **6** and diazo compound **9** at 75 °C for 3 h followed by desulfurization of the resulting episulfide with PPh_3_ to provide the overcrowded alkene **10**. After deprotection of the phenol groups, the attachment of two linker chains using 6‐bromohexan‐1‐ol was performed in the presence of a base and TBAI and the resulting motor **12** was treated with acryloyl chloride in the presence of base yielding the target bis‐acrylate motor molecule **M1**. Mono‐acrylate motor **M2,** not able to function as crosslinker, was obtained using a similar route (Figure 2 A). Enantiomeric pure (*R*)‐**M1** and (*S*)‐**M1** were obtained by preparative chiral HPLC. The separation and characterization of (*R*)‐**M1** and (*S*)‐**M1** are detailed in the Supporting Information.

### Rotary Cycle of Molecular Motor

A second‐generation molecular motor undergoes a full 360‐degree rotary cycle of the upper rotor part with respect to the lower stator part with the central olefinic double bond functioning as the rotational axle (Figure 2 A). The 4‐step rotary cycle involves two photoinduced isomerization processes (step 1 and 3) around the central double bond each followed by a thermal helix inversion (THI) step (step 2 and 4). Upon irradiation with UV‐light (*λ*_max_=365 nm) of **M1**, the stable *E*‐isomer [(*R*, *M*)‐stable‐*E*‐**M1**] is converted to an unstable isomer in which the methyl group at the stereogenic center is forced to adopt an energetically less‐favored pseudo‐equatorial orientation with a change in helicity of the molecule, resulting in (*R*, *P*)‐unstable‐*Z*‐**M1** isomer (Figure 2 A). A subsequent thermal helix inversion step results in release of the structural strain providing (*R*, *M*)‐stable‐*Z*‐**M1**, with the methyl group at the stereocenter in a more favorable pseudoaxial orientation and an inversion of the helicity from *P* to *M*. Another set of photochemical and thermal isomerization processes completes the 360° rotary cycle (Figure 2 A). Figure 2 B and C show the associated UV/Vis spectra of **M1** and **M2** in DCM solution at 263 K; both spectra displayed bathochromic shifts of the absorption upon irradiation at 365 nm with an increased band at 393 nm, which is characteristic of the formation of the unstable isomers (Figure 2 B,C).^[59]^ The samples were irradiated until no further change was observed and the photostationary states (PSS) were reached. Next the samples were kept in the dark at rt and the original spectra were regained as the result of the thermal helix inversion step. The kinetic studies (Figure S25 and S27) at different temperatures (*T*=283, 273, 263, 253 K) provided the rate constants of the first‐order thermal isomerization process, and the Gibbs energy of activation based on Eyring analysis (Figure S26 and S28), was 79.33 kJ mol^−1^ for the THI from unstable‐*Z* to stable‐*Z* isomer and 78.87 kJ mol^−1^ for THF from the unstable‐*E* to stable‐*E* isomer. The half‐lives (*t*_1/2_) were calculated to be 15.5 s and 12.9 s, respectively, for the unstable‐*E* and unstable‐*Z* isomers at room temperature. These values are similar to second‐generation motors with related core structures,^[59]^ indicating that the introduction of the functional groups has no significant influence on the overall rate‐determining thermal helix inversion step. The results confirm that the motor **M1** employed in the present study has a high rotary speed, considered crucial for the photo‐responsive behavior.

In addition, circular dichroism was used to confirm the photochemical and thermal steps of motor **M1**. (*R*)‐**M1** shows a negative CD absorption at 380 nm (Figure 2 D, black line) and upon irradiation with UV‐light a new positive CD band is observed at 420 nm, which is in accordance with the change of molecular helicity due to the formation of the unstable **M1** isomer (Figure 2 A, step 1). Independent study of enantiomer (*S*)‐**M1** showed similar but inverse CD effects (Figure 2 E). It should be noted that clear isosbestic points are maintained indicating unimolecular processes and keeping the samples in dark at rt (thermal helix inversion, Figure 2 A, step 2) after photo‐isomerization resulted in the original spectra in accordance with selective isomerization. And another cycle of irradiation and heating shows similar spectroscopic changes (Figure 2 A, step 3 and 4) indicative of the complete 360° rotary cycle and in accordance with our previous studies on second‐generation motors.^[37]^

### Bending and Walking Motion of Photo‐responsive LC Films

**Figure 3:**
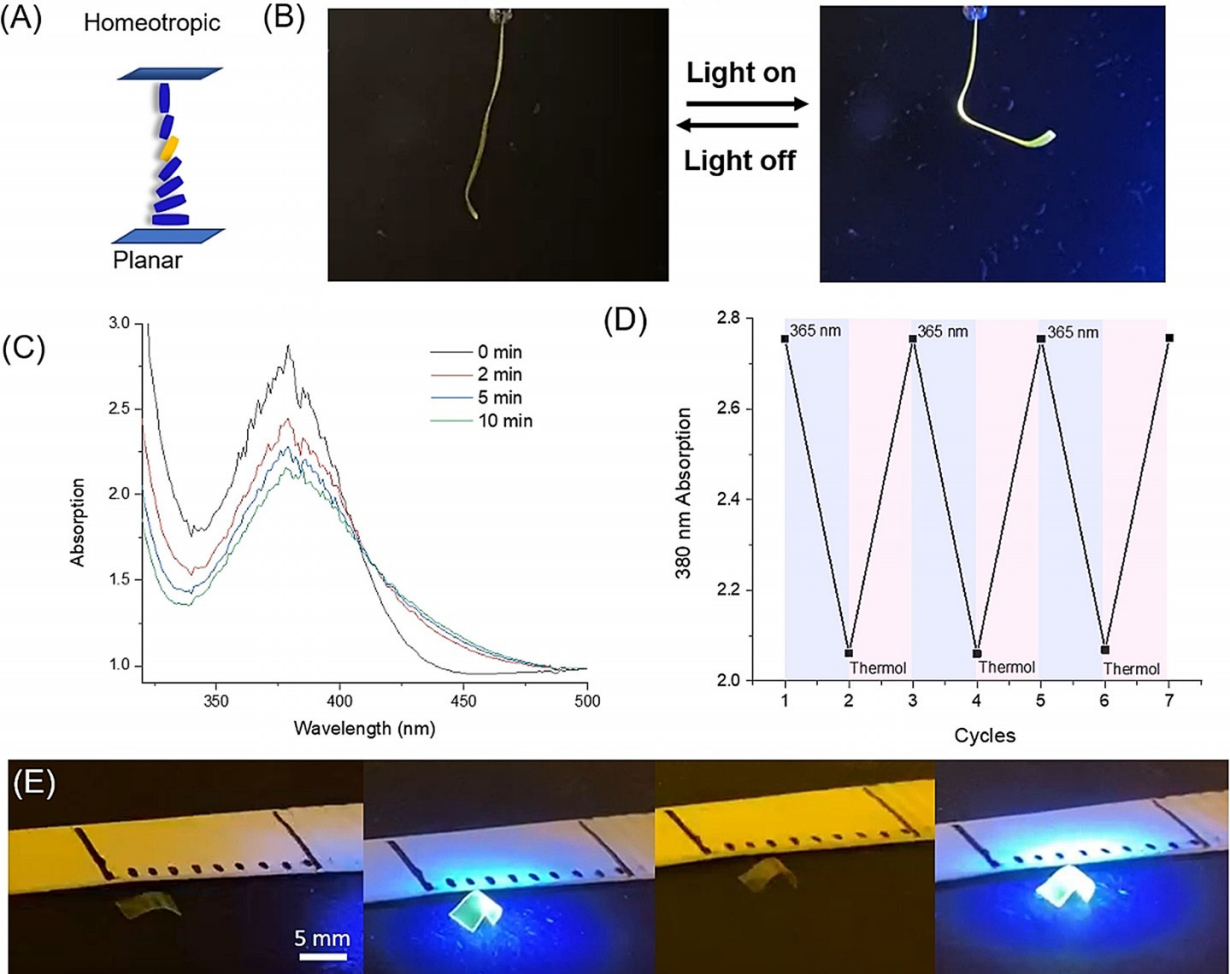
Photo‐triggered motion of LCN with racemic motors. A) Representative scheme of the LC alignment. Motor (orange) is mixed with LC monomers (blue). The mixture is aligned from homeotropic to planar in a splayed cell with a thickness of 25 μm. B) Bending of the ribbon upon UV irradiation (365 nm) (see also Supporting Movie S1). C) UV/Vis spectra of motor‐containing LC polymeric ribbon upon UV irradiation (365 nm). D) UV/Vis absorptions of the LC ribbon at 380 nm during the irradiation cycles. The ribbon is irradiated with the UV light (365 nm) ON and OFF for 6 cycles. E) Photo‐induced walking of an LC ribbon on a glass surface (Supporting Movie S2).

Following the characterization of the rotary motion of the motor in solution, the functioning of the motor as crosslinker and as a photo‐actuator in LCN thin films was studied. A mixture of 3 wt % racemic **M1** and LC monomers was stirred at 80 °C for 3 h prior to application. Before the curing process, the resulting mixture was introduced in splayed cells by capillary suction at 80 °C. We chose the splayed cell for the liquid‐crystal network (LCN) formation as splayed alignment usually gave rise to larger deformations of free‐standing films upon irradiation when compared to the samples with different alignments (uniaxial or parallel).^[45]^ The cells were subsequently cooled down to 45 °C and during this process, the doped liquid‐crystal monomer material undergoes a change from the isotropic phase to a nematic phase at around 60 °C (Figure S32) and the cell configuration enabled the LC mixture containing motor to align from homeotropic to parallel as shown in Figure 3 A. Next the mixture was copolymerized using blue (455 nm) light irradiation with a light intensity of 80 mw cm^−2^. The liquid‐crystal films were annealed at 125 °C for 10 min and cooled down to rt. The polarized optical microscopic (POM) images of the prepared films show a dark field when it was placed parallel to the cross‐polarized light and a bright field at 45° (Figure S33), which indicates that the polymeric LC films have a splayed alignment with a unidirectional projection of the orientation vector parallel to the rubbing direction of the lower substrate.[^45^, ^58^]

Following isolation of the polymer LC films from the cells, ribbons with a width of 3 mm were cut along the alignment direction at the planar orientation side of the sample. Irradiations were performed on free‐standing ribbons with UV light (365 nm) at an intensity of 100 mW cm^−2^ (see Supporting Information, for experimental set‐up). In the actuation study, the UV LED was placed at the homeotropic side of the film, the ribbons bend away from the light source (Figure 3 B). The ribbons could reach the saturated state in 2 s during illumination and recovered to their initial position instantaneously after switching off of light (Supporting Movie S1). The ribbon was studied with UV/Vis spectroscopy during the irradiation cycles.

Upon UV light (365 nm) illumination, the absorption of the film shows a decrease at 380 nm with a concomitant increase at 430 nm (Figure 3 C), which is similar to the change of the motor in solution (Figure 2 B). It indicates the rotary motion of the motor in the LC ribbon during the irradiation. When the UV‐light was switched off, the original spectra were regained. The photo‐actuation and recovery have been repeated several times and the system operates without any fatigue during the cycles (Figure 3 B,D, Supporting Movie S1). In order to explore the potential of the fast bending motion to induce translational motion,^[45]^ the LC film prepared by the above method was cut into a small piece with a length of 5 mm and placed on a flat glass surface. Upon irradiation, the LC film was able to walk a short distance in a direction towards the light source (Figure 3 E, Supporting Movie S2). For comparison, 3 wt % racemic **M2**, containing a single acrylate group, was used as dopant together with 18 wt % RM 23, 31 wt % RM 82, 46 wt % RM 105 and 2 wt % IRG 819. The control film was formed under identical conditions as those of **M1** using a splayed cell with a thickness of 25 μm, and also studied under the same photochemical conditions. However, no shape change was observed after UV light irradiation using this control compound not able to function as a cross‐linking unit in a liquid‐crystal network (Figure S30, Supporting Movie S3). It strongly supports the notion that the observed actuation of the LCN ribbon based on motor **M1** is predominantly due to the rotation and change in shape of the motor, and its effect on the order parameter of the LCN and cannot be attributed to a heating effect.^[58]^ The rotational motion of the motor, and its effect on the polymer main chains, reduce the order parameter of the mesogenic units which results in shrinkage along the molecular direction and expansion orthogonal to the ribbon. As a result of the applied splayed configuration the expansion difference at both sides of the film makes the film to bend.

### Helical Motion of Liquid‐Crystal Network

In order to study helical motion, we next used enantiomerically pure motors. We first tested the helical twisting power (HTP) of (*R*)‐**M1** and (*S*)‐**M1**. (*R*)‐**M1** and (*S*)‐**M1** were mixed with E7 respectively and the resulting mixtures were then filled into wedge cells by capillary force. The wedge cells were heated to 70 °C and then cooled down to room temperature, whereupon distinctive disclination lines were observed in the LC film through POM (Figure S31). The helical pitch measured by the Grandjean‐Cano method, was obtained using *P*=2 *R*tan*θ*, where *R* represents the distance between disclination lines and *θ* is the wedge angle of the wedge cells (tan*θ*=0.0078). With 1 wt % of (*R*)‐**M1** or (*S*)‐**M1** as a chiral dopant in E7, the helical pitch was determined to be 1.90±0.04 μm and 1.92±0.05 μm, respectively. This represents the helical twist power for both (*R*)‐**M1** and (*S*)‐**M1** being ±115 μm^−1^, which indicates **M1** being a strong chiral dopant.[^47^, ^54^, ^60^] Next the chiral doped LC material was prepared from (*R*)‐**M1** with the LC monomer mixture (18 wt % RM 23, 32 wt % RM 82, 46 wt % RM 105 and 2 wt % IRG 819) at 80 °C. The resulting LC mixture was filled in planar cells above the isotropic transition temperature and subsequently cooled to 40 °C to further process the mixture in its chiral‐nematic phase.

**Figure 4:**
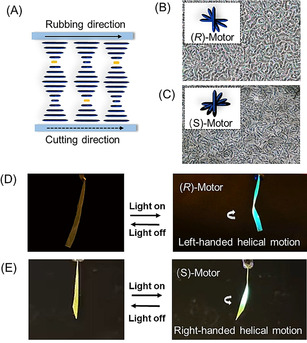
Photo‐triggered helical motion of LCN with enantiomeric pure motors. A) Representative scheme of the LC aliment. The motor (orange) was mixed with LC monomers (blue). The mixture was filled in a planar cell with a thickness of 25 μm and cured by blue light irradiation. B) POM image of the LC film with 1 wt % of (*R*)‐**M1**. C) POM image of the LC film with 1 wt % of (*S*)‐**M1**. D) LC ribbons with (*R*)‐**M1** showed left‐handed helical motion upon UV irradiation. E) LC ribbons with (*S*)‐**M1** showed right‐handed helical motion upon UV irradiation. All the ribbons were cut along the rubbing direction (see also Supporting Movies S4,S5).

To our delight, after curing with blue light, a structure with helical organization was obtained in the LC films (Figure 4 A) which was confirmed by the reflective colors in the POM images, although a lime structure is present that might point to some distortion of the helices from their pure planar organization (Figure 4 B). Furthermore, the enantiomeric form of the motor (*S*)‐**M1,** was also incorporated as a chiral dopant in the LCN and the twisted nematic phase was formed similar to that of the *R* enantiomer (Figure 4 C). Ribbons with a width of 3 mm were cut along the rubbing direction of the alignment layer. Next, the free‐standing ribbons were irradiated with 365 nm UV light at an intensity of 100 mW cm^−2^. The prepared LC films showed fast helical motion during the actuation experiment. Only 1 wt % (*R*)‐**M1** is needed to achieve left‐handed helical motion after irradiation (Figure 4 D, Supporting Movie S4) while the *S*‐enantiomer (*S*)‐**M1** displayed right‐handed motion (Figure 4 E, Supporting Movie S5). The ribbons were found to reach their saturated state in 2 s during illumination and recovered to their initial position as soon as the light was switched off (Figure 4 D, E; see also Supporting Movies S4, S5). Several cycles could be performed by subsequent irradiation and switching off of the light and the system did not show significant fatigue. Control experiments on two samples with opposite helices, due to the presence of enantiomeric chiral dopants as well as an achiral azobenzene photoswitch actuator, showed indeed twist effects upon irradiation with opposite helicity (Figure S34). This supports the molecular origin of the helicity change, the correlated gradient in stress and the chiral amplification from the molecular via the mesoscopic to the macromolecular level. In the present study, the fast helical motion is due to several unique features of these molecular rotary motors that are able to act as chiral dopant, responsive cross‐linker and photochemical ‐actuator in the LCN film. The motor is a strong chiral dopant when it was embedded in small amounts (1 wt %) in LC monomers and as a consequence induces the LC monomers to align in a helix structure. The approach presented here, taking advantage of the various intrinsic properties of a rotary motor in combination with controlled alignment, provides a facile method to obtain responsive materials to amplify and control several characteristic motions. The approach is distinctly different from earlier ways to achieve actuation involving LCN material which requires additional chiral dopant besides photochromic molecules to make controlled helix alignment in an LC film with twisted cell.^[57]^ Embedding all key features in a single motor, the change in helicity of the rotary motor, used as chiral dopant as well as cross‐linker, enables fast actuation and both left‐ and right‐handed helical twisting motions can be readily induced.

## Conclusion

Responsive soft polymeric materials which allow multiple distinct and well‐defined motions triggered by light are of major importance to enable the development of complex mechanical actuating systems. Here we show how a light‐driven molecular rotary motor incorporated in a liquid‐crystal (LC) polymer network can induce bending, walking and both left and right‐handed helical motion in the material.

A second‐generation molecular rotary motor functionalized with diacrylate moieties was incorporated as crosslinker in an LC‐polymer network without compromising its photochemical driven rotary motion. The motor unit has multiple functions that is, cross linker for the LC network, intrinsic chiral dopant and photo‐responsive units to allow autonomous motion upon irradiation with a single wavelength of light. Both racemic and homochiral motors were introduced in the LC network. The experimental data show that, in contrast to the racemic motor which did not affect the orientation of monomers in different alignments, the use of enantiomers resulted in the induction of different helical orientations of the LC monomers. The polymer ribbons with splayed alignment obtained from racemic motors show fast bending motion and surface walking upon irradiation. In contrast, the samples prepared with *R* and *S* chiral motors shown fast right‐handed or left‐handed helical motion, respectively, during illumination with UV light. Control experiments indicate that the sense of helicity induced in the polymer ribbons is governed by the intrinsic chirality of the motor dopant and the twisting motions are fully reversible following the isomerization steps and associated dynamic helicity change of the motor unit during the rotary cycle.

Distinct from other approaches to achieve the conversion of light energy in reversible helical motion and amplification of mechanical effects along length scales from the molecular to macroscopic level, all key functions are embedded in a single molecular structure. Using a molecular motor as crosslinker and chiral dopant in an LC‐network, taking advantage of the shape change, dynamic chirality and control of directionality of movement, complex mechanical responses including bending, walking and twisting motions are achieved. The ability to govern complex autonomous motions based on the delicate interplay of intrinsic photo‐responsive motor function, dynamic chirality and organization as shown here open new avenues for future smart materials.

## Conflict of interest

The authors declare no conflict of interest.

## Supporting information

As a service to our authors and readers, this journal provides supporting information supplied by the authors. Such materials are peer reviewed and may be re‐organized for online delivery, but are not copy‐edited or typeset. Technical support issues arising from supporting information (other than missing files) should be addressed to the authors.

Supplementary

Supplementary

Supplementary

Supplementary

Supplementary

Supplementary
