# Supplementary material for: Photo‐responsive Helical Motion by Light‐Driven Molecular Motors in a Liquid‐Crystal Network
Source: Angew Chem Int Ed Engl. 2021 Mar 12;60(15):8251–7. doi: 10.1002/anie.202016254 (PMC8048625; doi:10.1002/anie.202016254)
Supplement: Supplementary file 1 — Supplementary [file ANIE-60-8251-s005.pdf]

Angewandte Chemie
Eine Zeitschrift der Gesellschaft Deutscher Chemiker

Supporting Information

Photo-responsive Helical Motion by Light-Driven Molecular Motors in a Liquid-Crystal Network

Jiaxin Hou, Anirban Mondal, Guiying Long, Laurens de Haan, Wei Zhao, Guofu Zhou, Danqing Liu, Dirk J. Broer, Jiawen Chen, and Ben L. Feringa**

anie_202016254_sm_miscellaneous_information.pdf
anie_202016254_sm_movie_S1.mp4
anie_202016254_sm_movie_S2.mp4
anie_202016254_sm_movie_S3.mp4
anie_202016254_sm_movie_S4.mp4
anie_202016254_sm_movie_S5.mp4

Supporting Information

Experimental Section

General remarks

Chemicals were purchased from Acros, Aldrich, Fluka or Merck and were used as received. Solvents for extraction and chromatography were technical grade. All solvents used in reactions were freshly distilled from appropriate drying agents before use. All reactions were performed under inert atmosphere (Ar). Analytical TLC was performed with Merck silica gel 60 F254 plates and visualization was accomplished by UV light. Flash chromatography was carried out using Merck silica gel 60 (230-400 mesh ASTM). Solvents for spectroscopic studies were of spectrophotometric grade (UVASOL Merck). Vertically aligned polyimide (PI; DL-4018) was purchased from Shenzhen Dalton Electronic Materials Coo., Ltd..

Characterizations: ^{1}H-NMR spectra were recorded on a Bruker AVANCE NEO (600 MHz), a Varian AMX-400 (400 MHz). The corresponding chemical shifts were reported in δ values (ppm) relative to deuteron chloroform ($CDCl_3$, ^{1}H δ=7.25, ^{13}C δ=77.2) For ^{1}H-NMR, the signals were assigned as following: singlet (s), doublet (d), double doublet (dd), triplet (t), quartet (q) and multiplet (m). Proton magnetic resonance spectroscopy (HMRS) was measured using a double focusing high-resolution mass spectrometer (MS-902, AEI). Ultraviolet-visible (UV-Vis) spectra were obtained with SPECORD_S600 spectrophotometer in a 1 cm quartz cuvette. Solution circular dichroism (CD) spectra were recorded on a JASCO J-715 spectropolarimeter. Irradiation experiments were performed using an M365L2-C1-365 nm, 120 mW (Typ.) collimated LED (Throlabs). All the optical phenomena of LCN were observed and recorded via polarizing optical microscope (POM; DM2700p, Leica). Separation and characterization of the enantiomers of **M1** was achieved by a Shimadzu LC-10ADVP HPLC using chiral stationary phase (Chiralpak AD).

Preparation of planar and splayed cell

For the preparation of the planar cell, a glass substrate was thoroughtly cleaned and spin-coated with polyvinyl achcohol solution (5 wt.% PVA in water). After removing all the water at 100 °C, the substrate was rubbed at velvet to form the alignment layer. Another cover glass plate with the same planar alignment layer was sticked together parallely to the substarte and fixed by the UV-curing spacer (Suzhou Nanomicro Technology Co., Ltd), to construct the LC cell.

For the preparation of the splayed cell, a glass substrate was thoroughtly cleaned and spin-coated with Polyimide alignment layer. The glass plate was then heated at 180 °C for 1.5 h to remove the solvent. Another cover glass plate with planar alignment layer was sticked together with a spacing distance of 25 μm fixed by the UV-curing spacer (Suzhou Nanomicro Technology Co., Ltd), to construct the LC cell.

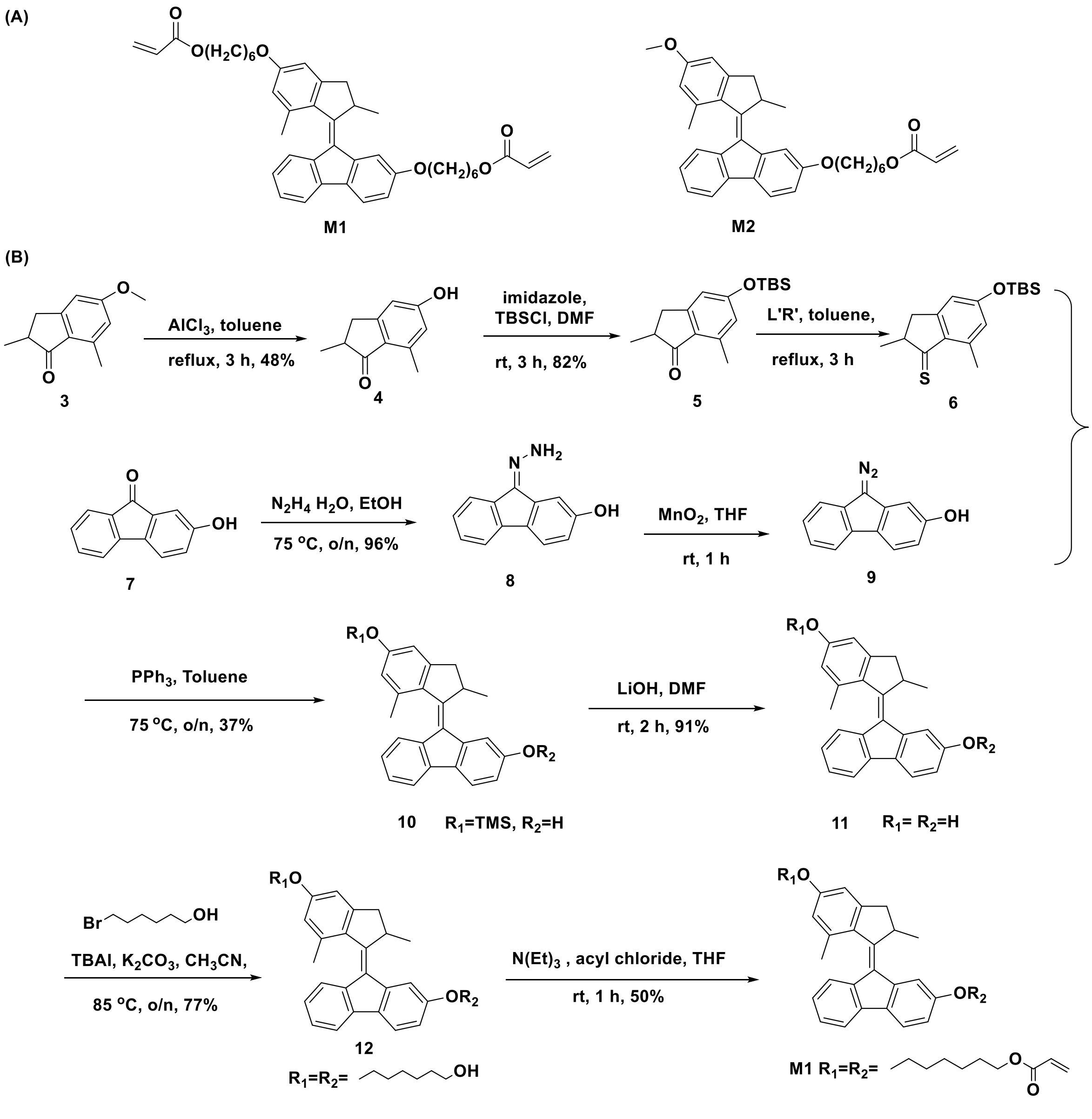

Scheme S1. Light-driven rotary molecular motors. (a) Structure of acrylate functionalized motors **M1** (bis-functionalized) and **M2** (mono-functionalized**)**. (b) Synthetic route of **M1**.

5-methoxy-2,7-dimethyl-2,3-dihydro-1H-inden-1-one (3)

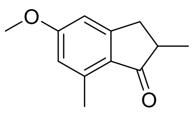

1-methoxy-3-methylbenzene (5.20 mL, 41 mmol) was added to mechanically stirred polyphosphoric acid (50 g) at 70 °C. After 10 min, methacrylic acid (5.20 mL, 60 mmol) was added slowly and the mixture was stirred at 70 °C for 4 h. After the mixture was cooled down to room temperature, Water (300 mL) was added. The mixture was extracted with EtOAc (2 × 150 mL). The combined organic layers were washed with brine and dried over Na_2SO_4. The solvent was removed in vacuo and the residue was recrystallized twice with pentane to yield ketone **3** as white solids (1.30 g, 6.80 mmol, 17%). ^{1}H NMR (600 MHz, Chloroform-*d*) δ 7.55 (s, 1H), 6.83 (s, 1H), 3.93 (d, *J* = 1.1 Hz, 3H), 3.34 (d, *J* = 9.2 Hz, 1H), 2.78 – 2.56 (m, 2H), 2.24 (s, 3H), 1.45 – 1.18 (m, 3H). ^{13}C NMR (151 MHz) δ 154.6, 127.3, 125.5, 106.4, 77.3, 77.0, 76.8, 55.6, 42.1, 35.0, 16.7, 16.4. HRMS (ESI) calcd for $C_{12}H_{14}O_2$ 191.1067 [M+], found 191.1068.

5-hydroxy-2,7-dimethyl-2,3-dihydro-1H-inden-1-one (4)

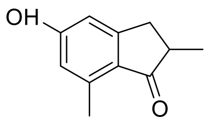

To a solution of ketone **2** (0.50 g, 2.60 mmol) in dry toluene (20 mL), $AlCl_3$ (1.73g, 13 mmol) was added. The mixture was stirred at reflux for 3 hours under nitrogen. After cooling down to room temperature, the mixture was quenched with water. The mixture was extracted with EtOAc (2 ×100 mL). The combined organic layers were washed with brine and dried over Na_2SO_4. The solvent was removed with reduced pressure. The residue was purified by flash column (SiO_2, pentane: EtOAc=5:1) to yield ketone **4** (0.22 g, 1.25 mmol, 48%) as white solids. ^{1}H NMR (600 MHz, DMSO-d_6) δ 10.46 (s, 1H), 6.85 (s, 1H), 3.23 (dd, *J* = 16.5, 7.3 Hz, 1H), 2.73 – 2.36 (m, 2H), 2.14 (s, 3H), 1.14 (d, *J* = 7.2 Hz, 3H). ^{13}C NMR (151 MHz, DMSO-d_6) δ 206.8, 162.7, 154.5, 127.9, 125.7, 125.3, 111.4, 41.7, 34.4, 16.8, 16.5. HRMS (ESI) calcd for $C_{11}H_{12}O_2$ 177.0871 [M+], found 177.0875

5-((tert-butyldimethylsilyl)oxy)-2,7-dimethyl-2,3-dihydro-1H-inden-1-one (5)

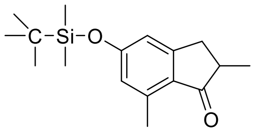

To a mixture of **3** (0.37 g, 2.10 mmol) and imidazole (0.50 g, 7.10 mmol) in dry DMF (20 mL), *tert*-Butyldimethylsilyl chloride (0.86 g, 5.68 mmol) was added. The mixture was stirred at room temperature overnight under nitrogen, then quenched with water. The mixture was extracted with EtOAc (2 × 100 mL). The organic layer was washed with brine and dried over Na_2SO_4. The solvent was removed in vacuo. The residue was purified by flash column (SiO_2, pentane: EtOAc=8:1) to yield **5** (0.50 g, 1.72 mmol, 82%) as a colorless

oil. ^{1}H NMR (400 MHz, Chloroform-*d*) δ 6.61 (s, 1H), 6.53 (s, 1H), 3.22 (dd, *J* = 16.6, 7.5 Hz, 1H), 2.67 – 2.58 (m, 1H), 2.54 (s, 3H), 1.24 (d, *J* = 7.1 Hz, 3H), 0.96 (s, 9H), 0.21 (s, 6H). ^{13}C NMR (101 MHz, Chloroform-*d*) δ 211.3, 163.8, 159.5, 143.6, 130.4, 124.2, 116.9, 45.1, 37.2, 28.3, 28.2, 21.0. HRMS (ESI) calcd for $C_{17}H_{26}O_2Si$ 291.1775 [M+], found 291.1778.

(2-((tert-butyldimethylsilyl)oxy)-9H-fluoren-9-ylidene)hydrazine (8)

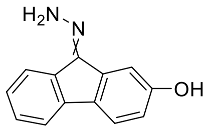

To a solution of **7** (0.70 g, 2.26 mmol) in EtOH (50 mL), hydrazine (20 mL, 22.60 mmol) was added. The mixture was stirred at 75 °C overnight and the solvent was evaporated. water (200 mL) was added to the mixture. The crude product was extracted with EtOAc (2×150 mL). The combined organic layer was washed with brine and dried over Na_2SO_4. The solvent was removed with reduced pressure. The residue was purified by flash column (SiO_2, pentane: EtOAc=1:1) to yield **8** (0.70 g, 2.16 mmol, 96%) as yellow solid. ^{1}H NMR (600 MHz, DMSO-d_6) δ 9.62 (s, 1H), 9.50 (s, 1H), 8.09 (d, *J* = 7.6 Hz, 1H), 7.94 (s, 1H), 7.84 (s, 2H), 7.72 (d, *J* = 7.5 Hz, 1H), 7.69 (d, *J* = 8.1 Hz, 1H), 7.64 (dd, *J* = 7.5, 0.9 Hz, 1H), 7.60 – 7.58 (m, 1H), 7.58 – 7.54 (m, 1H), 7.37 – 7.33 (m, 1H), 7.25 (dtd, *J* = 7.4, 3.7, 1.2 Hz, 2H), 7.17 (td, *J* = 7.4, 1.1 Hz, 1H), 7.03 (d, *J* = 2.2 Hz, 1H). ^{13}C NMR (151 MHz, DMSO-d_6) δ 158.0, 158.0, 131.6, 128.9, 127.6, 126.3, 126.3, 125.4, 121.4, 121.2, 119.8, 119.5, 119.0, 115.6, 115.1, 113.4, 106.7. HRMS (ESI) calcd for $C_{13}H_{10}N_2O$ 211.0827 [M+], found 211.0829.

(*E*,*Z*)-tert-butyl((9-(5-((tert-butyldimethylsilyl)oxy)-2,7-dimethyl-2,3-dihydro-1H-inden-1-ylidene)-9H-fluoren-2-yl)oxy)dimethylsilane (*E*-10, *Z*-10)

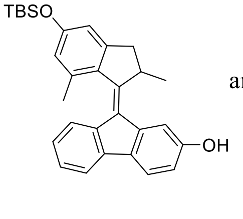

and

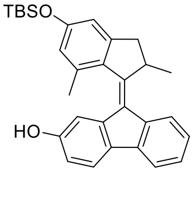

To a solution of **5** (0.50 g, 1.72 mmol) in dry toluene (50 mL), Lawesson's reagent (1.39 g, 3.44 mmol) was added. The mixture was stirred at reflux for 2 h and the solvent was evaporated. The residue was purified by flash column (SiO_2, pentane: EtOAc=10:1) to obtain a purple solution of thioketone **6.** The solvent was removed in reduced pressure to obtain thioketone **6** as purple solid. Thioketone was immediately added to a solution of diazo compound **9** in dry THF (10 mL). The diazo compound was prepared by mixing MnO_2 (1.20 g, 13.40 mmol) and hydrazone **8** (0.70 g, 2.16 mmol) in THF (10 mL) for 2 h and followed by a filtration to remove the MnO_2. The diazo and thioketone mixture was

stirred at 75 °C for 3 h. After 3 h, PPh_3 (0.70 g, 2.60 mmol) was added to the mixture. The mixture was stirred at 75 °C overnight and the solvent was removed in vacuo. The residue was purified by flash column (SiO_2, pentane: EtOAc=10:1) to yield motor *E*-**10** (0.25 g, 0.44 mmol, 26%) and motor *Z*-**10** (0.15 g, 0.26 mmol, 15%) as a yellow solid. *E*-**10:** ^{1}H NMR (600 MHz, Chloroform-*d*) δ 8.01 (s, 1H), 7.94 (d, *J* = 2.3 Hz, 1H), 7.83 (d, *J* = 7.7 Hz, 1H), 7.78 – 7.73 (m, 1H), 7.70 (dd, *J* = 7.4, 1.4 Hz, 1H), 7.63 (d, *J* = 8.2 Hz, 1H), 7.32 (d, *J* = 1.0 Hz, 1H), 7.30 – 7.26 (m, 1H), 6.83 – 6.82 (m, 1H), 4.11 – 4.04 (m, 1H), 3.32 (dd, *J* = 14.9, 6.0 Hz, 1H), 2.57 (d, *J* = 14.7 Hz, 1H), 2.28 (s, 3H), 1.30 (d, *J* = 6.4Hz, 3H), 1.08 (s, 9H), 0.34 (dd, *J* = 7.2, 2.8 Hz, 6H). HRMS (ESI) calcd for $C_{30}H_{34}O_2Si$ 455.2362 [M+], found 455.2381.

Z-**10:** ^{1}H NMR (600 MHz, Chloroform-*d*) δ 8.38 (d, *J* = 7.9 Hz, 1H), 8.01 (s, 1H), 7.66 (dd, *J* = 11.4, 7.8 Hz, 2H), 7.38 (d, *J* = 2.2 Hz, 1H), 7.28 (s, 1H), 7.13 – 7.09 (m, 1H), 6.85 (dd, *J* = 8.2, 2.2 Hz, 1H), 6.82 (s, 1H), 4.05 – 4.00 (m, 1H), 3.32 (dd, *J* = 14.8, 6.0 Hz, 1H), 2.57 (d, *J* = 14.8 Hz, 1H), 2.26 (s, 3H), 1.30 (d, *J* = 6.7 Hz, 3H), 1.08 (s, 9H), 0.33 (d, *J* = 6.6 Hz, 6H). HRMS (ESI) calcd for $C_{30}H_{34}O_2Si$ 455.2362 [M+], found 455.2379.

9-(5-hydroxy-2,7-dimethyl-2,3-dihydro-1H-inden-1-ylidene)-9H-fluoren-2-ol (11)

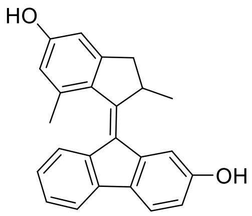

To a solution of motor *E*-**10** (0.40g, 0.70mmol) in DMF (10 mL), LiOH (0.084 g, 3.50 mmol) was added. The mixture was stirred at room temperature for 2 h and quenched with water. The pH value of the mixture was adjusted to 6 with HCl (1 M) solution. The water phase was extracted with EtOAc (2 × 150 mL). The combined organic layers were washed with brine and dried over Na_2SO_4. The residue was purified by flash column (SiO_2, pentane: EtOAc=1:1) to yield **7** (0.22 g, 0.63 mmol, 91%) as yellow solid. ^{1}H NMR (600 MHz, DMSO-*d*$_6$) δ 9.84 (s, 1H), 9.53 (s, 1H), 9.33 (s, 1H), 7.75 – 7.71 (m, 1H), 7.70 – 7.66 (m, 1H), 7.25 (ddd, *J* = 11.9, 7.2, 1.4 Hz, 1H), 7.21 (t, *J* = 6.9 Hz, 1H), 6.79 (dd, *J* = 8.2, 2.1 Hz, 1H), 6.74 (ddd, *J* = 14.6, 9.1, 2.2 Hz, 2H), 6.62 (dd, *J* = 9.9, 2.2 Hz, 1H), 3.96 (dd, *J* = 11.4, 6.3 Hz, 1H), 3.21 (ddd, *J* = 15.7, 10.4, 5.9 Hz, 1H), 2.13 (d, *J* = 17.3 Hz, 3H), 1.17 (t, *J* = 7.1 Hz, 3H).^{13}C NMR (151 MHz, DMSO-*d*$_6$) δ 170.8, 152.6, 152.6, 139.6, 131.0, 127.1, 126.9, 125.9, 125.3, 123.6, 123.0, 121.0, 120.6, 119.0, 118.7, 116.2, 115.9, 114.8, 114.4, 110.9, 110.9, 110.3, 60.2, 44.7, 44.5,

41.4, 41.4, 40.4, 40.3, 40.1, 39.9, 39.8, 39.7, 39.6, 21.7, 21.7, 21.2, 19.4, 19.4, 14.5. HRMS (ESI) calcd for $C_{24}H_{20}O_2$ 341.1536 [M+], found 341.1545.

6-((9-(5-((6-hydroxyhexyl)oxy)-2,7-dimethyl-2,3-dihydro-1H-inden-1-ylidene)-9H-fluoren-2-yl)oxy)hexan-1-ol (12)

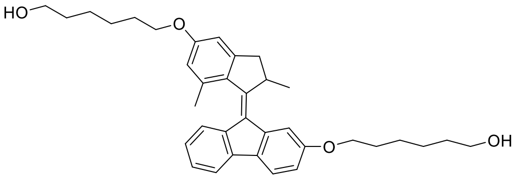

To a solution of **11** (0.1 g, 0.29 mmol) in CH_3CN (10 mL), K_2CO_3 (0.20 g, 1.45 mmol), TBAI (0.086 g, 0.23 mmol) and 6-bromohexan-1-ol (0.15 mL, 1.16 mmol) were added. The mixture was stirred at 85 ^{o}C under nitrogen overnight. The solvent was removed in reduced pressure. The residue was dissolved in EtOAc (50 mL). The organic layer was washed with water (2×100 mL) and brine, then dried over Na_2SO_4. The solvent was removed in vacuo and the residue was purified by flash column (SiO_2, pentane: EtOAc=1:3) to yield motor **12** (0.12 g, 0.22 mmol, 77%). ^{1}H NMR (600 MHz, Chloroform-*d*) δ 7.84 (d, *J* = 7.6 Hz, 1H), 7.74 – 7.69 (m, 1H), 7.66 (dd, *J* = 16.2, 7.8 Hz, 1H), 7.33 (td, *J* = 7.4, 1.1 Hz, 1H), 7.31 – 7.27 (m, 1H), 7.01 (d, *J* = 2.3 Hz, 1H), 6.87 (dd, *J* = 8.3, 2.3 Hz, 1H), 6.83 – 6.81 (m, 1H), 6.71 (dd, *J* = 5.2, 2.3 Hz, 1H), 4.17 – 4.03 (m, 4H), 3.86 (tdd, *J* = 9.3, 6.6, 2.7 Hz, 1H), 3.73 – 3.64 (m, 4H), 3.33 (dt, *J* = 14.7, 5.1 Hz, 1H), 2.58 (dd, *J* = 14.8, 2.4 Hz, 1H), 2.28 (d, *J* = 26.7 Hz, 3H), 1.88 (dt, *J* = 8.2, 6.4 Hz, 3H), 1.80 – 1.72 (m, 1H), 1.68 – 1.53 (m, 10H), 1.54 – 1.39 (m, 6H), 1.34 (dd, *J* = 9.7, 6.7 Hz, 3H). ^{13}C NMR (151 MHz, DMSO-d_6) δ 159.4, 150.9, 138.5, 131.9, 125.7, 125.5, 124.6, 124.3, 122.5, 122.2, 119.1, 118.8, 117.8, 117.4, 113.7, 113.6, 112.0, 109.3, 108.1, 108.0, 76.2, 76.0, 75.8, 67.3, 67.0, 67.0, 66.9, 61.9, 61.8, 61.8, 43.6, 43.6, 40.6, 31.7, 31.7, 31.6, 28.4, 28.3, 28.3, 25.0, 24.9, 24.9, 24.6, 24.5, 24.5, 20.7, 20.7, 17.9, 17.8. HRMS (ESI) calcd for $C_{36}H_{44}O_4$ 541.3273 [M+], found 541.3281.

6-((9-(5-((6-(acryloyloxy)hexyl)oxy)-2,7-dimethyl-2,3-dihydro-1H-inden-1-ylidene)-9H-fluoren-2-yl)oxy)hexyl acrylate (M1)

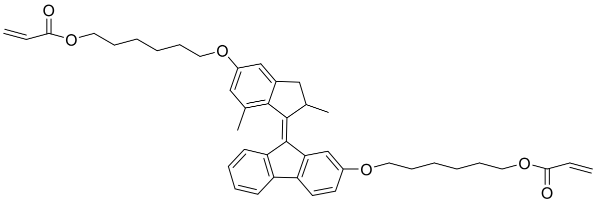

To a solution of **12** (0.10 g, 0.22 mmol) in THF (5 mL), triethylamine (0.61 mL, 4.4 mmol) and acryloyl chloride (0.04 mL, 0.49 mmol) were added at 0 ^{o}C. The mixture was allowed to warm up to room temperature and stirred for 2 h. The mixture was quenched with water (100 mL) and the aqueous phase was

extracted with EtOAc (2 × 150 mL). The combined organic layer was washed with saturated NH_4Cl solution (2 × 150 mL) and brine, then dried over Na_2SO_4. The solvent was removed in vacuo and the residue was purified by flash column (SiO_2, pentane: EtOAc=6:1) to yield motor **M1** (0.07 g, 0.11 mmol, 50%). The separation and characterization were performed by HPLC using chiral stationary phase (Chiralpak AD, heptane : isopropanol 90 : 10) with a flow rate of 1.0 mL/min (Figure S29). We compared the CD spectra with the reported CD spectra of a motor with similar structure and assigned the *R* and *S* enantiomers.[1]

^{1}H NMR (400 MHz, Chloroform-*d*) δ 7.81 (d, *J* = 7.5 Hz, 1H), 7.74 – 7.58 (m, 2H), 7.37 – 7.17 (m, 3H), 6.97 (d, *J* = 2.3 Hz, 1H), 6.84 (dd, *J* = 8.3, 2.3 Hz, 1H), 6.79 (s, 1H), 6.68 (d, *J* = 2.3 Hz, 1H), 6.46 – 6.35 (m, 2H), 6.20 – 6.05 (m, 2H), 5.82 (tt, *J* = 8.0, 1.8 Hz, 2H), 4.29 – 3.97 (m, 8H), 3.84 (tt, *J* = 6.5, 3.3 Hz, 1H), 3.31 (dt, *J* = 14.6, 4.8 Hz, 1H), 2.56 (dd, *J* = 14.9, 2.1 Hz, 1H), 2.27 (s, 2H), 1.85 (h, *J* = 6.1 Hz, 3H), 1.81 – 1.64 (m, 6H), 1.64 – 1.38 (m, 8H), 1.32 (t, *J* = 6.9 Hz, 3H). ^{13}C NMR (151 MHz, Chloroform-*d*) δ 166.4, 166.4, 158.7, 158.7, 153.5, 153.5, 148.7, 148.5, 139.6, 132.9, 132.8, 130.7, 130.6, 130.6, 129.8, 129.6, 128.6, 127.4, 126.8, 126.4, 125.6, 125.0, 124.3, 124.0, 123.9, 123.5, 123.0, 120.0, 119.9, 118.7, 118.6, 115.1, 112.7, 110.9, 108.3, 107.8, 107.8, 68.2, 68.0, 67.9, 67.8, 67.8, 64.6, 64.6, 60.4, 42.8, 41.0, 40.9, 35.0, 34.5, 31.5, 31.5, 30.3, 30.1, 29.7, 29.6, 29.3, 29.2, 29.2, 28.6, 28.6, 28.5, 25.9, 25.9, 25.9, 25.8, 25.8, 25.8, 25.6, 21.1, 19.4, 19.3, 16.7, 16.6, 14.2. HRMS (ESI) calcd for $C_{42}H_{48}O_6$ 649.3523 [M+], found 649.3518.

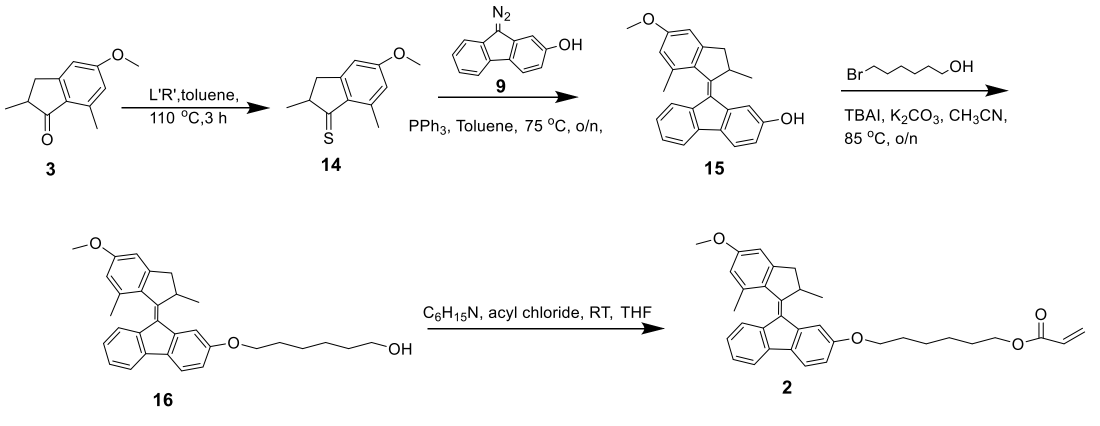

Figure S1 Synthetic route of **M2**

Tert-butyl((9-(5-methoxy-2,7-dimethyl-2,3-dihydro-1H-inden-1-ylidene)-9H-fluoren-2-yl)oxy)dimethylsilane (15)

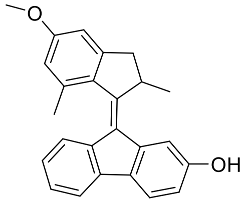

To a solution of **3** (0.13 g, 0.75 mmol) in dry toluene (5 mL), Lawesson's reagent (0.60g, 1.50 mmol) was added. The mixture was stirred at reflux for 2 h and the solvent was evaporated. The residue was purified by flash column (SiO_2, pentane: DCM=3:1) to obtain the purple solution of thioketone **14.** The solvent was removed in reduced pressure to obtain thioketone **14** as purple solid. Thioketone was immediately added to a solution of diazo compound **9** in dry THF (10 mL). The diazo compound was prepared by mixing MnO_2 (0.18 g, 2.15 mmol) and hydrazone **8** (0.14 g, 0.43 mmol) in THF (10 mL) for 2 h and followed by a filtration to remove the MnO_2. The diazo and thioketone mixture was stirred at 75 ^oC for 3 h. After 3 h, PPh_3 (0.15 g, 0.57 mmol) was added to the mixture. The mixture was stirred at 75 ^oC overnight and the solvent was removed in vacuo. The residue was purified by flash column (SiO_2, pentane: EtOAc=4:1) to yield motor **15** (0.05 g, 0.11 mmol, 26%) as yellow solid. ^1H ^1H NMR (600 MHz, Chloroform-*d*) δ 7.84 (d, *J* = 7.5 Hz, 1H), 7.70 – 7.61 (m, 2H), 7.38 – 7.27 (m, 2H), 7.10 (td, *J* = 7.6, 1.2 Hz, 1H), 6.91 – 6.78 (m, 2H), 6.71 (dd, *J* = 8.3, 2.4 Hz, 1H), 4.15 – 4.06 (m, 1H), 3.90 (d, *J* = 11.1 Hz, 3H), 3.40 – 3.26 (m, 1H), 2.58 (dd, *J* = 14.6, 8.8 Hz, 1H), 2.36 – 2.25 (m, 3H), 1.36 – 1.31 (m, 3H). ^{13}C NMR (151 MHz, Chloroform-*d*) δ 160.9, 155.3, 155.2, 152.1, 149.4, 139.3, 139.0, 137.8, 133.1, 133.0, 128.6, 126.8, 126.6, 125.7, 125.3, 123.5, 123.3, 120.4, 120.1, 118.8, 118.3, 114.2, 114.2, 114.1, 113.9, 110.9, 110.1, 108.8, 108.7, 68.0, 55.4, 55.4, 44.8, 44.6, 41.6, 41.6, 25.6, 21.7, 21.7, 18.9. HRMS (ESI) calcd for $C_{25}H_{22}O_2$ 355.1653 [M+], found 355.1648.

6-((9-(5-methoxy-2,7-dimethyl-2,3-dihydro-1H-inden-1-ylidene)-9H-fluoren-2-yl)oxy)hexan-1-ol (16)

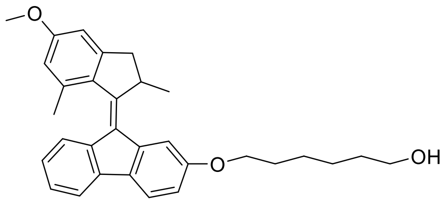

To a solution of **15** (0.05 g, 0.14 mmol) in CH_3CN (5 mL), K_2CO_3 (0.08 g, 0.58 mmol), TBAI (0.04 g, 0.11 mmol) and 6-bromohexan-1-ol (0.15 mL, 0.77 mmol) were added. The mixture was stirred at 85 ^oC under nitrogen overnight. The solvent was removed in reduced pressure. The residue was dissolved in EtOAc (30 mL). The organic layer was washed with water (2×50 mL) and brine, then dried over Na_2SO_4. The solvent was removed in vacuo and the residue was purified by flash column (SiO_2, pentane: EtOAc=2:1) to yield motor **16** (0.05 g, 0.11 mmol, 79%) as yellow oil.

^{1}H NMR (600 MHz, Chloroform-*d*) δ 7.77 – 7.64 (m, 2H), 7.48 (d, *J* = 2.3 Hz, 1H), 7.41 (d, *J* = 7.8 Hz, 1H), 7.36 – 7.26 (m, 2H), 7.16 – 7.11 (m, 1H), 6.94 (dd, *J* = 8.3, 2.2 Hz, 1H), 6.89 (dd, *J* = 8.3, 2.3 Hz, 1H), 6.85 (d, *J* = 2.9 Hz, 1H), 6.75 (dd, *J* = 6.9, 2.4 Hz, 1H), 4.09 (t, *J* = 6.5 Hz, 1H), 3.90 (d, *J* = 2.5 Hz, 3H), 3.71 – 3.62 (m, 3H), 3.42 (t, *J* = 6.7 Hz, 1H), 3.38 – 3.32 (m, 1H), 2.61 (dd, *J* = 14.8, 6.8 Hz, 1H), 2.36 (s, 2H), 2.31 (s, 4H), 1.92 – 1.86 (m, 2H), 1.77 (t, *J* = 7.2 Hz, 1H), 1.68 – 1.55 (m, 6H), 1.53 – 1.45 (m, 4H), 1.41 – 1.37 (d, 3H), 1.36 (d, 1H). ^{13}C NMR (151 MHz, Chloroform-*d*) δ 160.9, 158.8, 151.9, 149.4, 141.3, 139.6, 139.4, 138.9, 137.9, 133.1, 133.0, 128.9, 126.9, 126.7, 125.8, 125.4, 123.6, 123.3, 120.2, 119.9, 118.9, 118.5, 114.3, 114.2, 114.2, 113.1, 110.5, 109.2, 108.8, 108.6, 68.4, 68.1, 62.8, 62.7, 62.6, 55.4, 55.4, 44.8, 44.7, 41.7, 33.9, 32.8, 32.7, 32.7, 32.5, 29.5, 29.3, 28.0, 26.1, 26.0, 25.7, 25.6, 25.0, 21.8, 21.8, 19.0, 18.9. HRMS (ESI) calcd for $C_{31}H_{34}O_3$ 455.2541 [M+], found 455.2547.

6-((9-(5-methoxy-2,7-dimethyl-2,3-dihydro-1H-inden-1-ylidene)-9H-fluoren-2-yl)oxy)hexyl acrylate (M2)

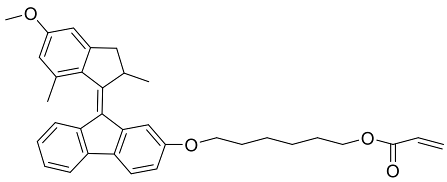

To a solution of **16** (0.03g, 0.07 mmol) in THF (5 mL), triethylamine (0.61 mL, 4.4 mmol) and acryloyl chloride (0.04 mL, 0.49 mmol) were added at 0 °C. The mixture was allowed to warm up to room temperature and stirred for 2 h. The mixture was quenched with water (50 mL) and the aqueous phase was extracted with EtOAc (2 × 100 mL). The combined organic layer was washed with saturated NH_4Cl solution (2 × 100 mL) and brine, then dried over Na_2SO_4. The solvent was removed in vacuo and the residue was purified by flash column (SiO_2, pentane: EtOAc=8:1) to yield motor **2** (0.02 g, 0.04 mmol, 60%) as yellow oil. ^{1}H NMR (400 MHz, Chloroform-*d*) δ 7.79 – 7.59 (m, 2H), 7.35 – 7.19 (m, 2H), 7.07 (dd, *J* = 7.6, 1.2 Hz, 1H), 6.93 – 6.76 (m, 2H), 6.68 (d, *J* = 2.4 Hz, 1H), 6.39 (ddd, *J* = 17.3, 3.0, 1.5 Hz, 1H), 6.11 (ddd, *J* = 17.3, 10.4, 2.6 Hz, 1H), 5.80 (dt, *J* = 10.4, 1.7 Hz, 1H), 4.24 – 4.00 (m, 4H), 3.87 (d, *J* = 1.6 Hz, 3H), 3.38 – 3.24 (m, 1H), 2.56 (dd, *J* = 14.8, 2.2 Hz, 1H), 2.26 (d, *J* = 16.2 Hz, 3H), 1.85 (dd, *J* = 8.3, 6.5 Hz, 1H), 1.80 – 1.64 (m, 3H), 1.65 – 1.37 (m, 4H), 1.31 (t, *J* = 6.8 Hz, 3H).^{13}C NMR (101 MHz, Chloroform-*d*) δ 169.0, 163.6, 163.5, 161.4, 161.3, 154.5, 154.4, 152.1, 152.0, 143.9, 142.4, 142.2, 142.0, 141.6, 141.3, 140.5, 135.7, 135.7, 135.6, 135.3, 133.2, 133.1, 131.5, 131.4, 131.2, 129.4, 129.2, 128.3, 128.0, 126.1, 125.9, 122.8, 122.4, 121.5, 121.0, 116.8, 116.7, 115.7, 113.0, 111.7, 111.3, 111.2, 80.0, 79.7, 79.4, 70.9, 70.6, 67.2, 58.0,

58.0, 47.3, 47.3, 44.3, 31.9, 31.8, 31.2, 31.2, 28.5, 28.4, 28.4, 28.4, 24.4, 24.4, 21.6, 21.5. HRMS (ESI) calcd for $C_{34}H_{36}O_4$ 509.2686 [M+], found 509.2673.

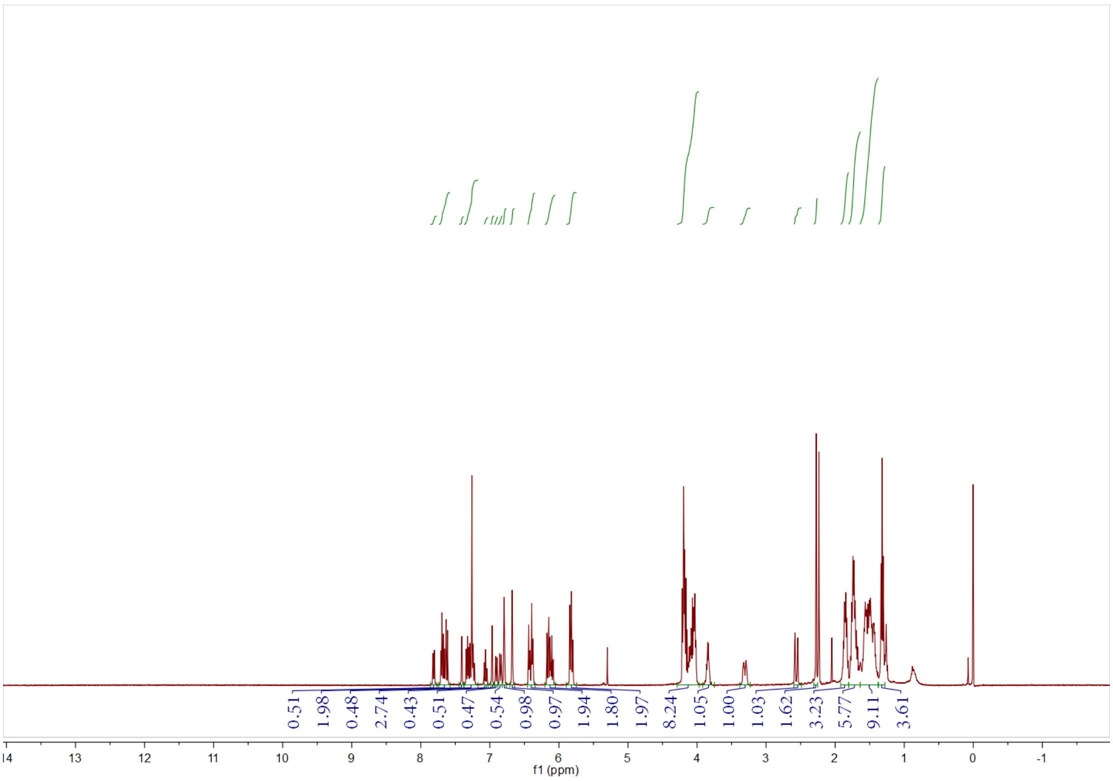

Figure S2 ^{1}H-NMR spectrum of **M1**

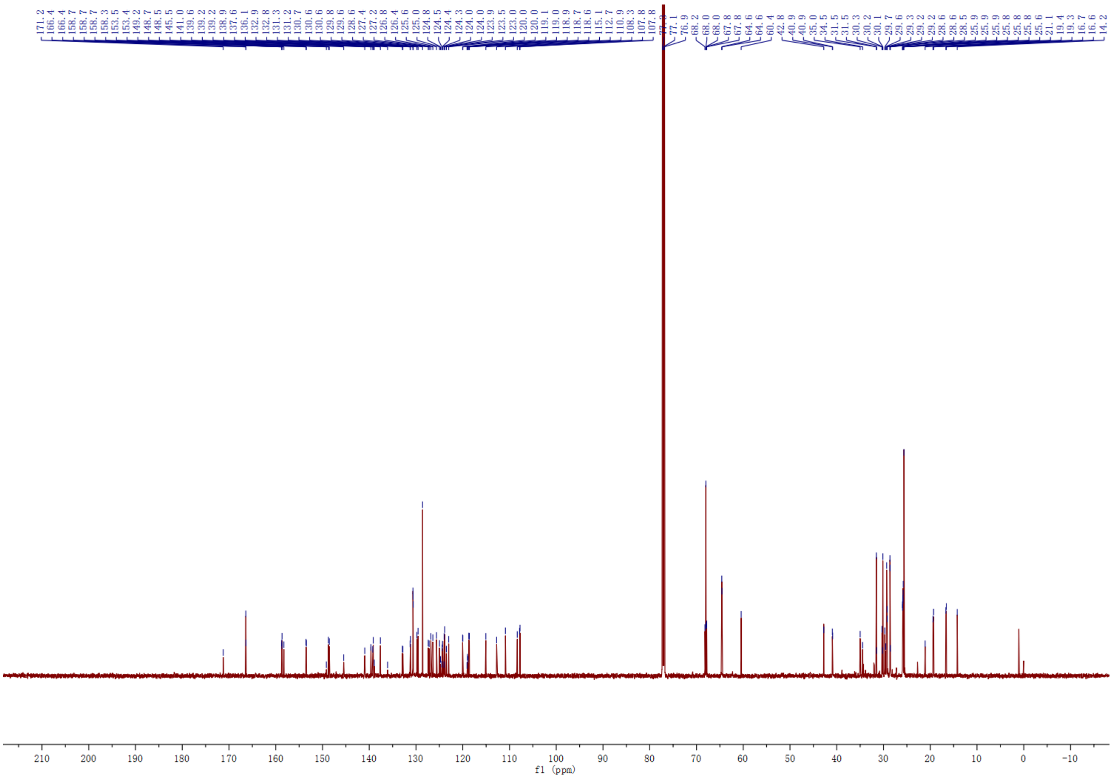

Figure S3 ^{13}C-NMR spectrum of **M1**

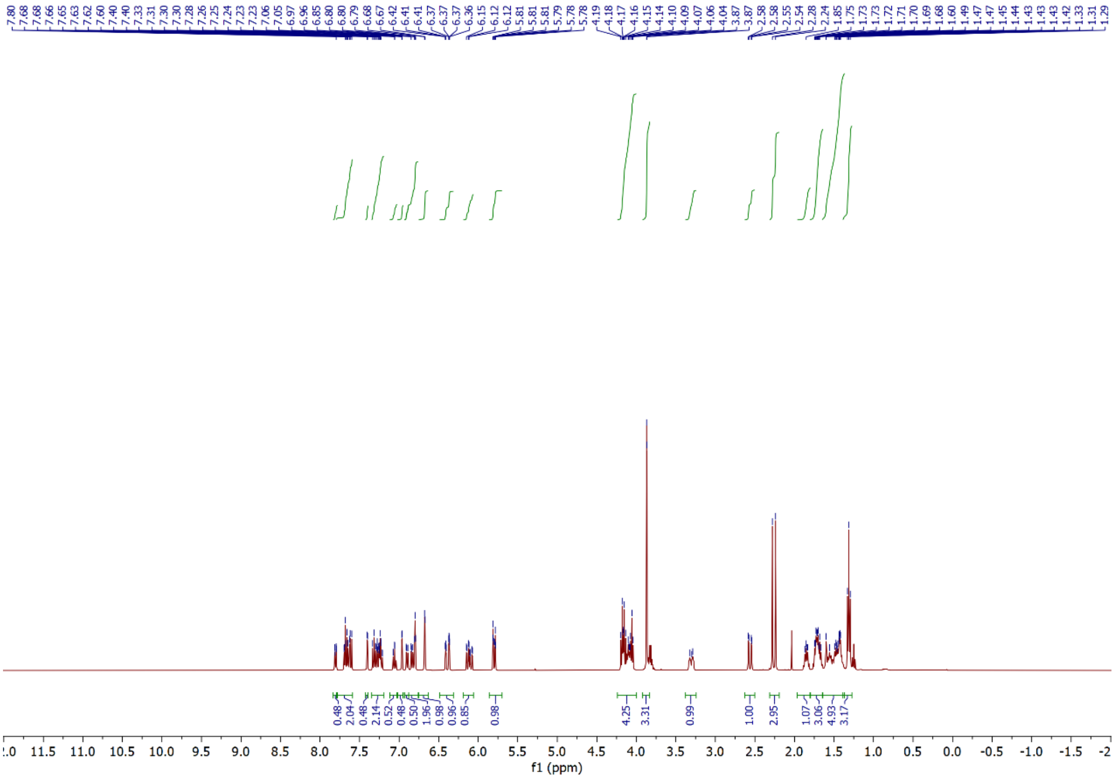

Figure S4 ^{1}H-NMR spectrum of **M2**

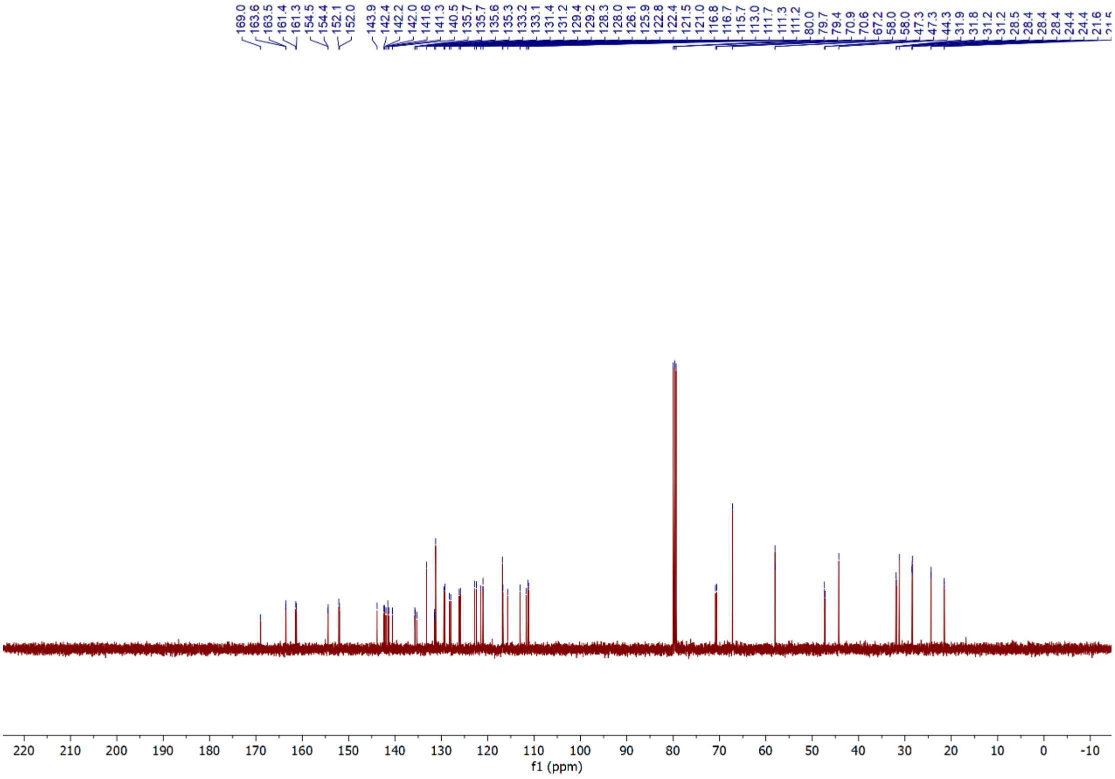

Figure S5 ^{13}C-NMR spectrum of M2

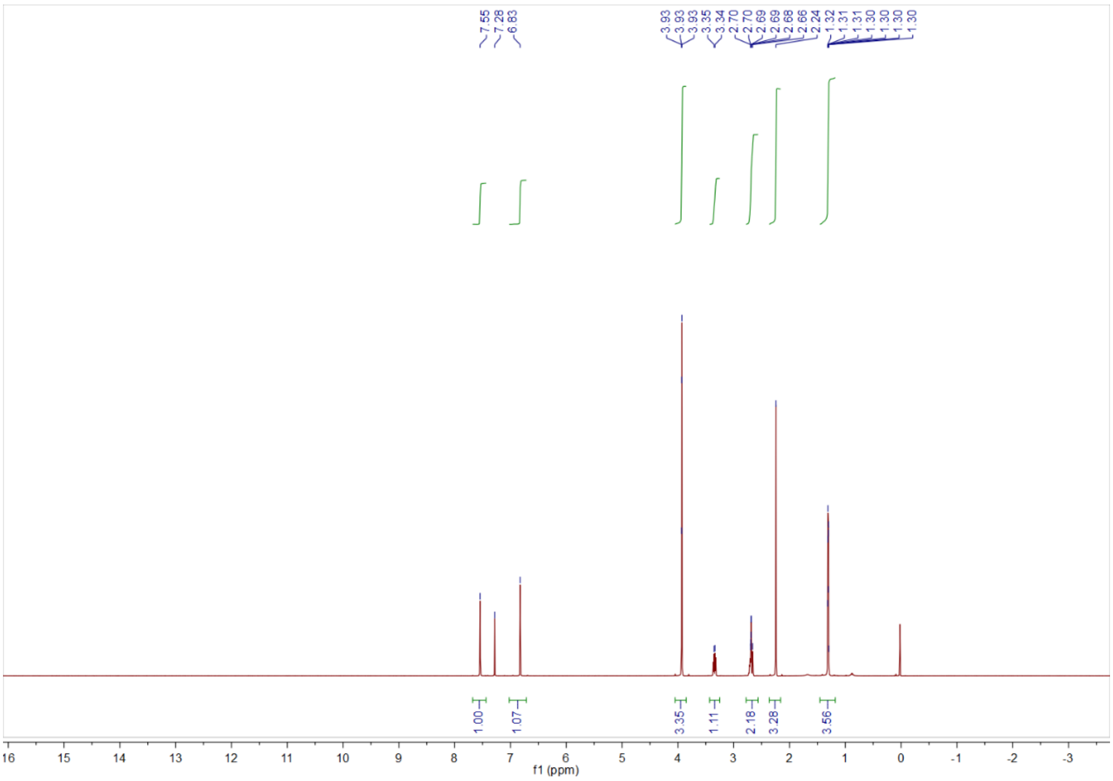

Figure S6 ^{1}H-NMR spectrum of **compound 3**

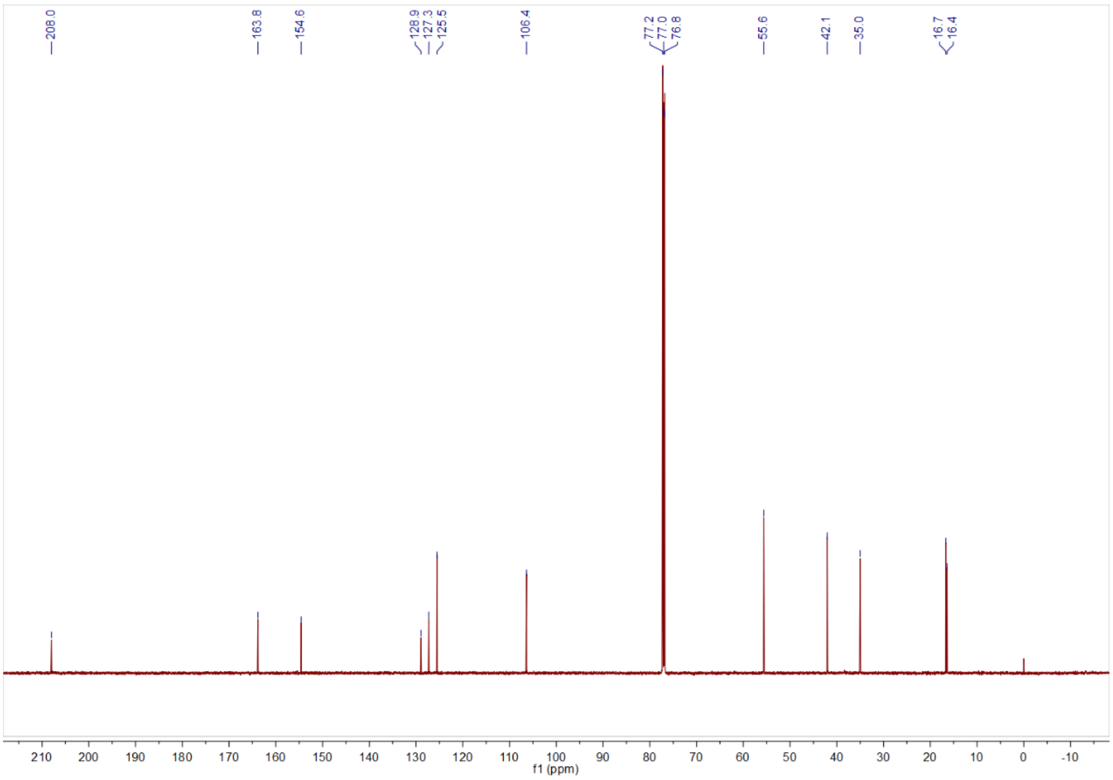

Figure S7 ^{13}C-NMR spectrum of compound 3

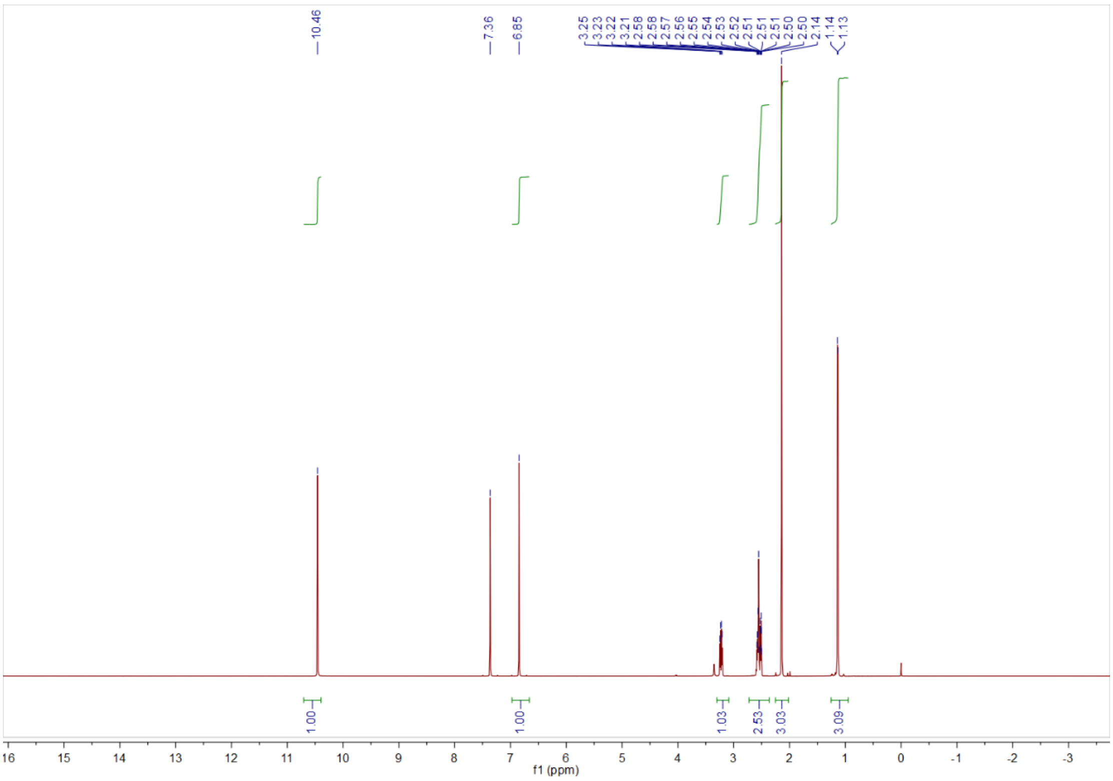

Figure S8 ^{1}H-NMR spectrum of **compound 4**

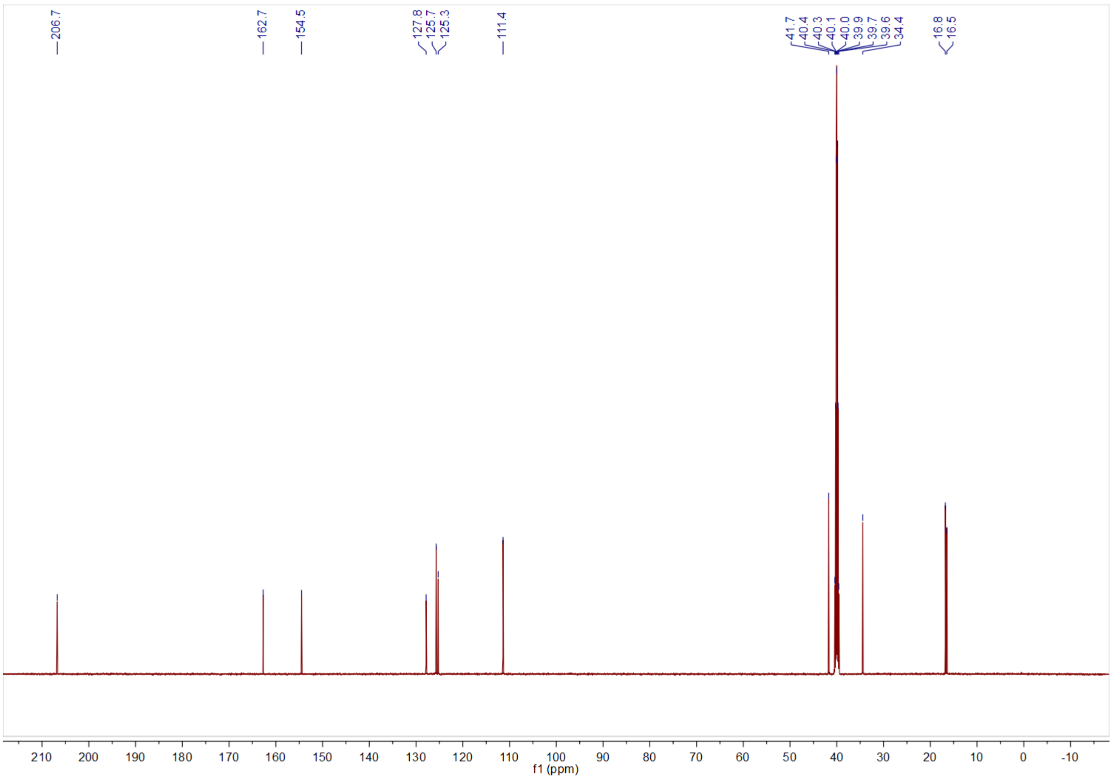

Figure S9 ^{13}C-NMR spectrum of compound 4

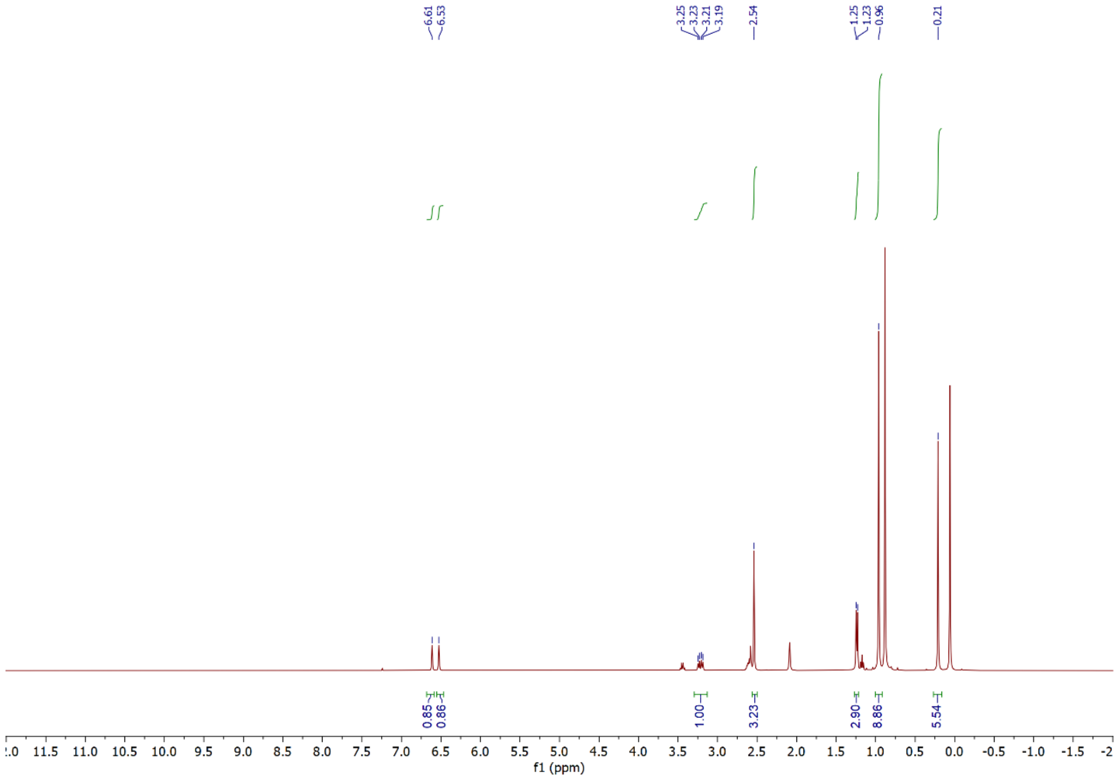

Figure S10 ^{1}H-NMR spectrum of compound 5

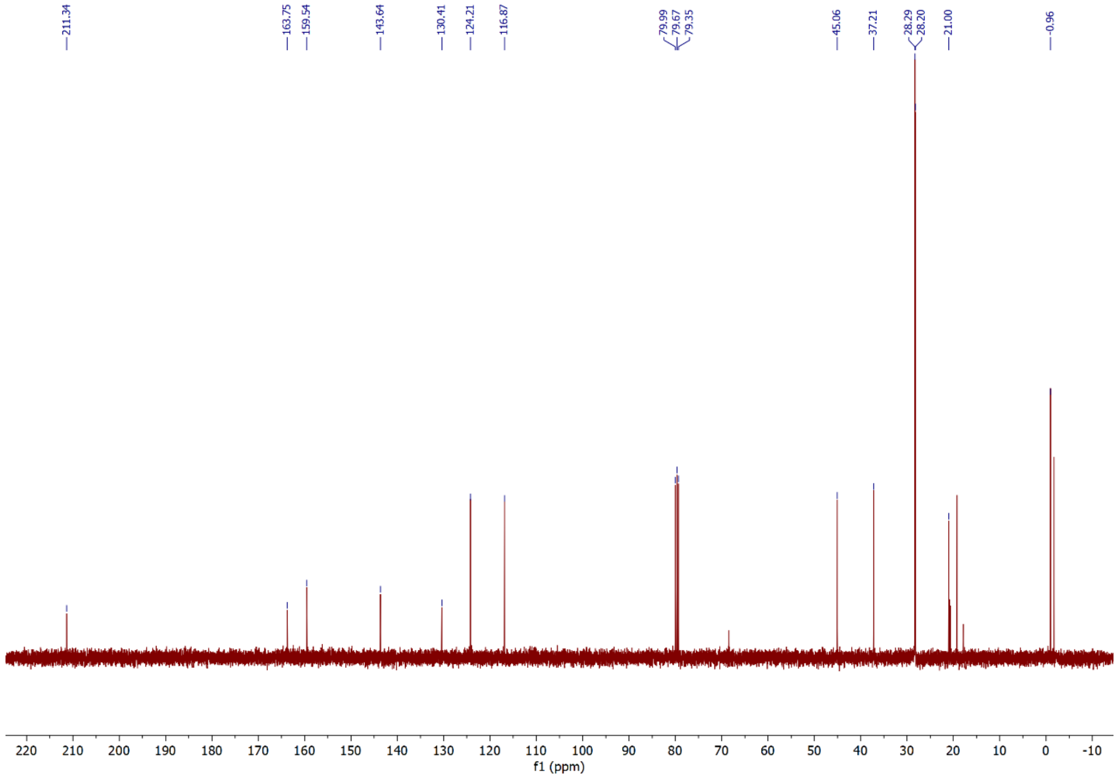

Figure S11 ^{13}C-NMR spectrum of compound 5

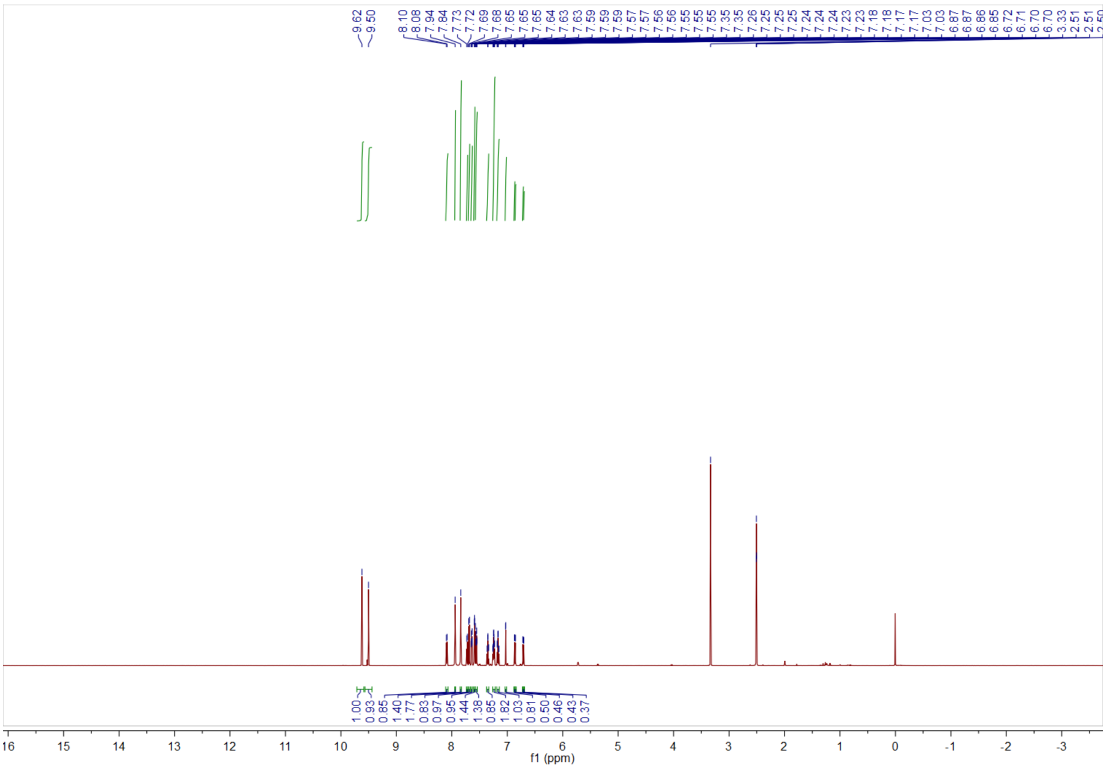

Figure S12 ^{1}H-NMR spectrum of **compound 8**

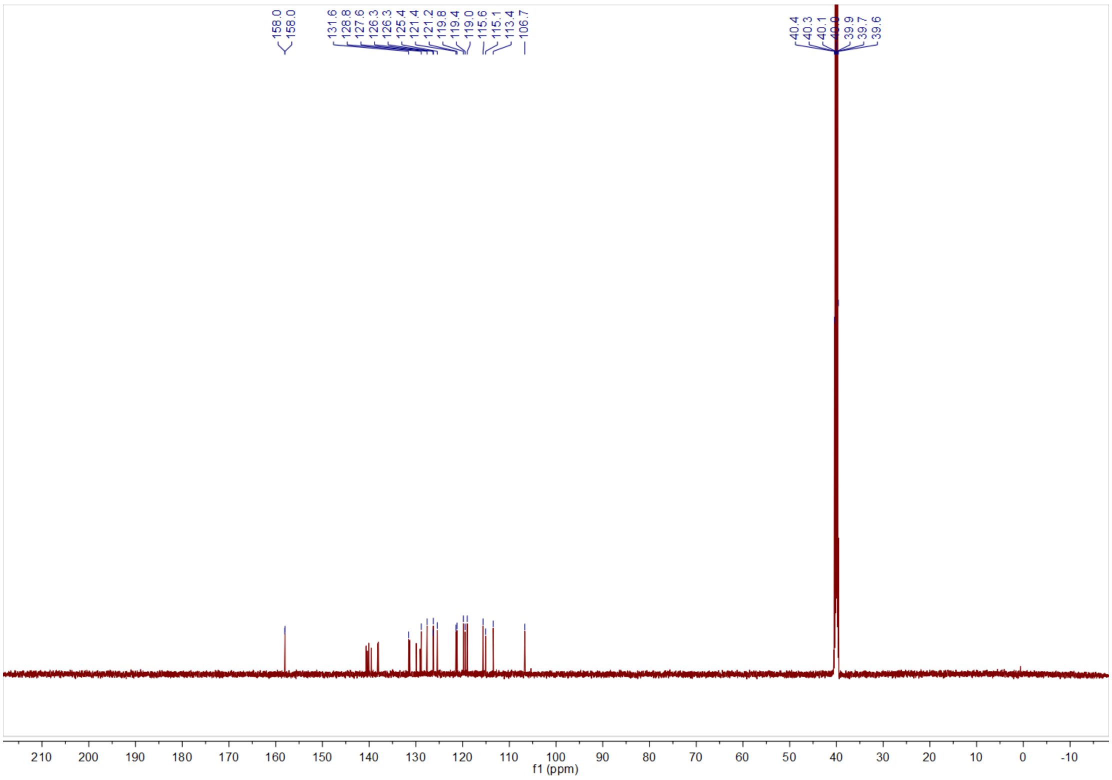

Figure S13 ^{13}C-NMR spectrum of compound 8

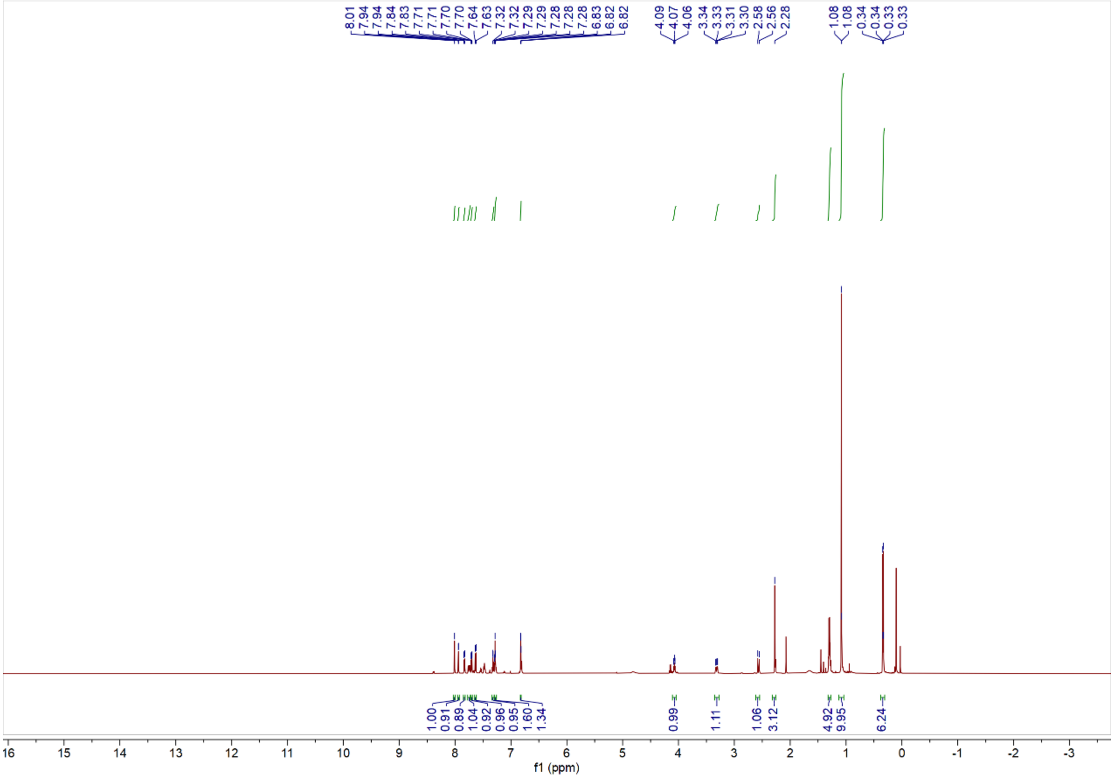

Figure S14 ^{1}H-NMR spectrum of **compound E-10**

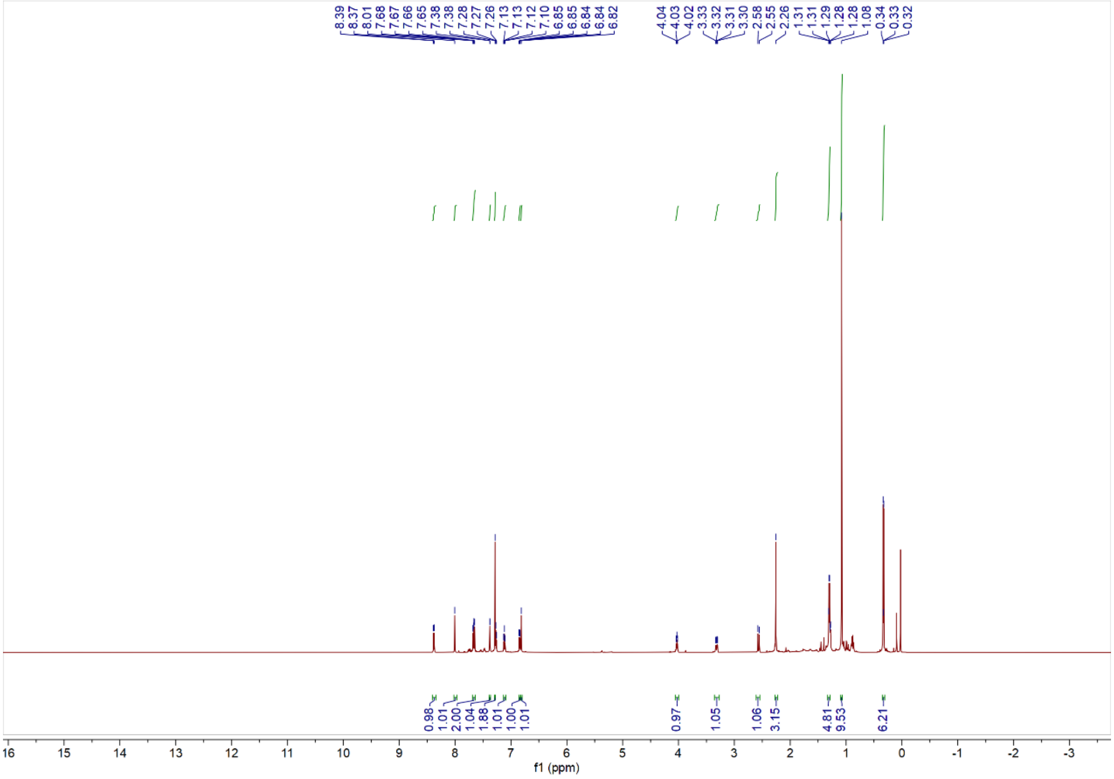

Figure S15 ^{1}H-NMR spectrum of **compound Z-10**

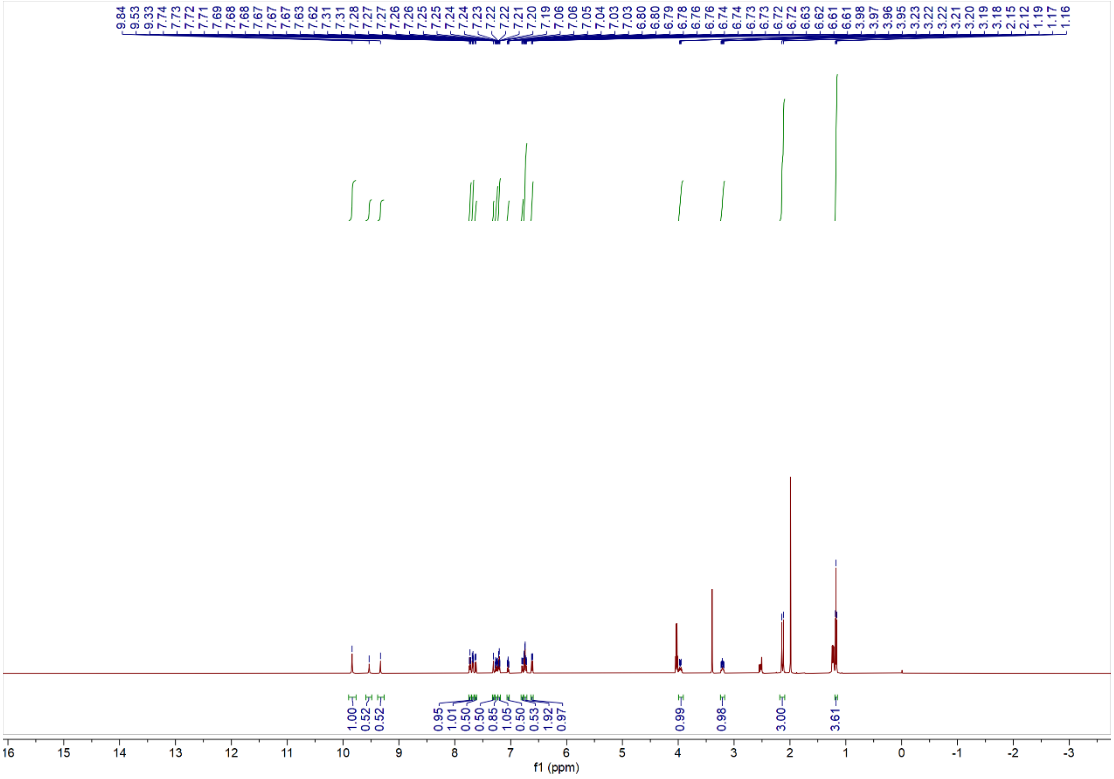

Figure S16 ^{1}H-NMR spectrum of **compound 11**

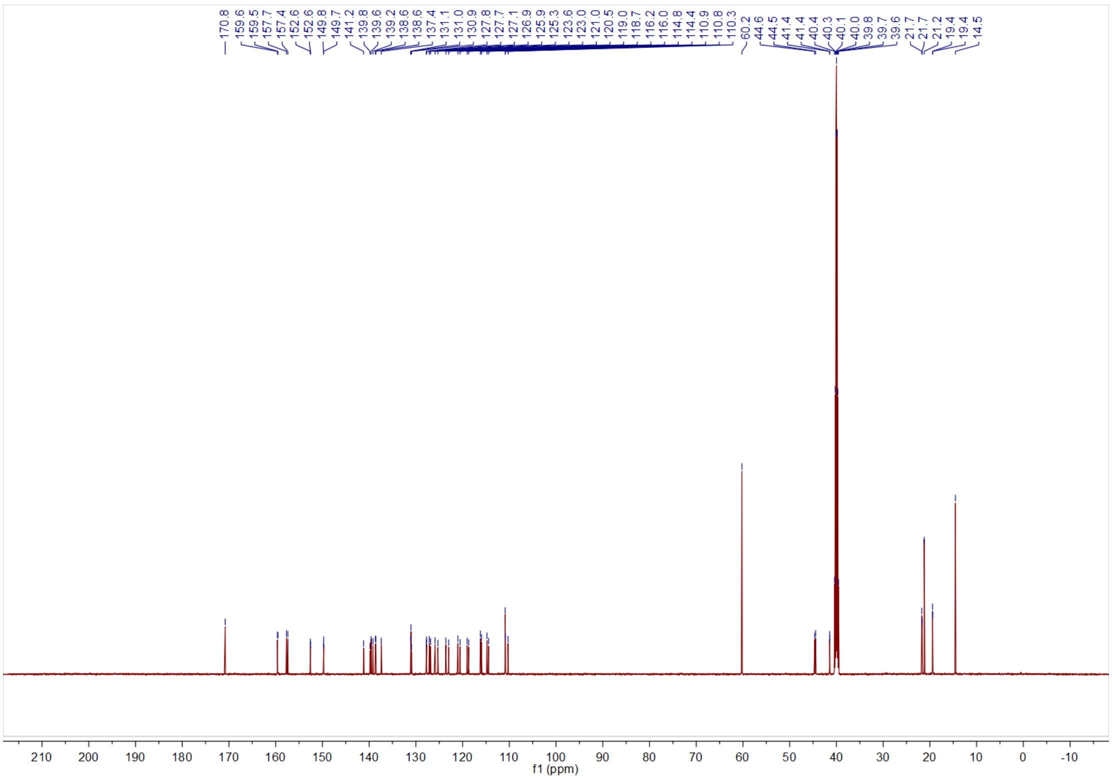

Figure S17 ^{13}C-NMR spectrum of compound 11

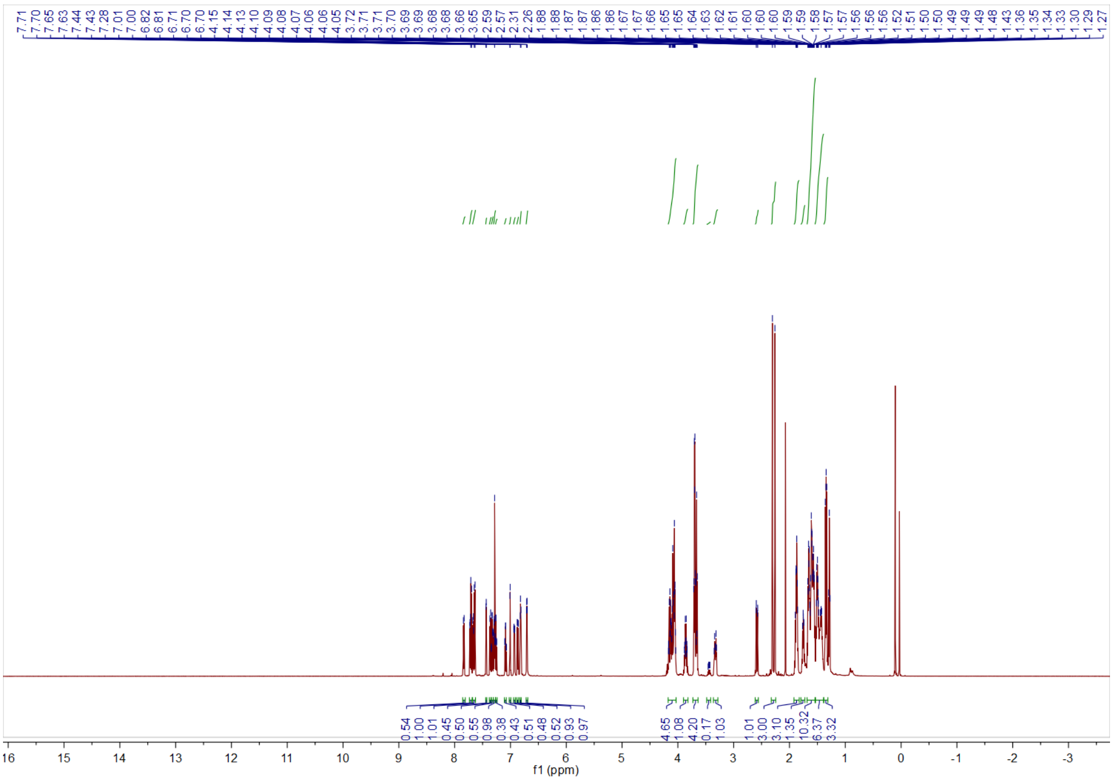

Figure S18 ^{1}H-NMR spectrum of **compound 12**

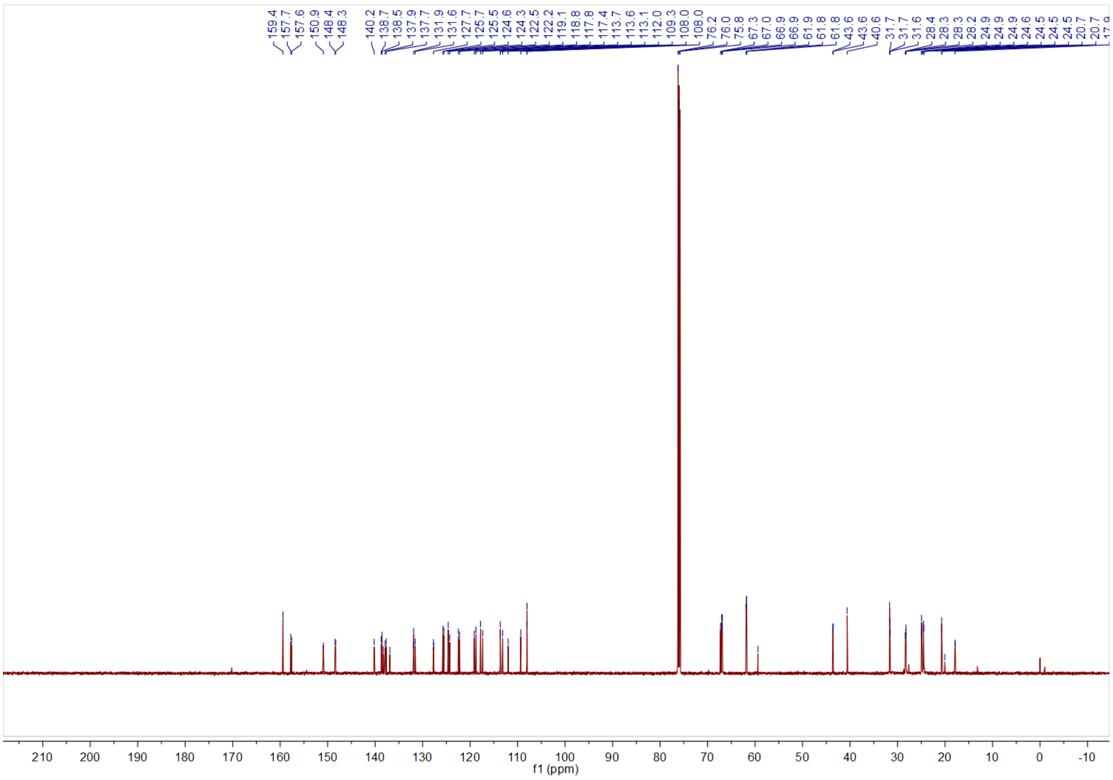

Figure S19 ^{13}C-NMR spectrum of compound 12

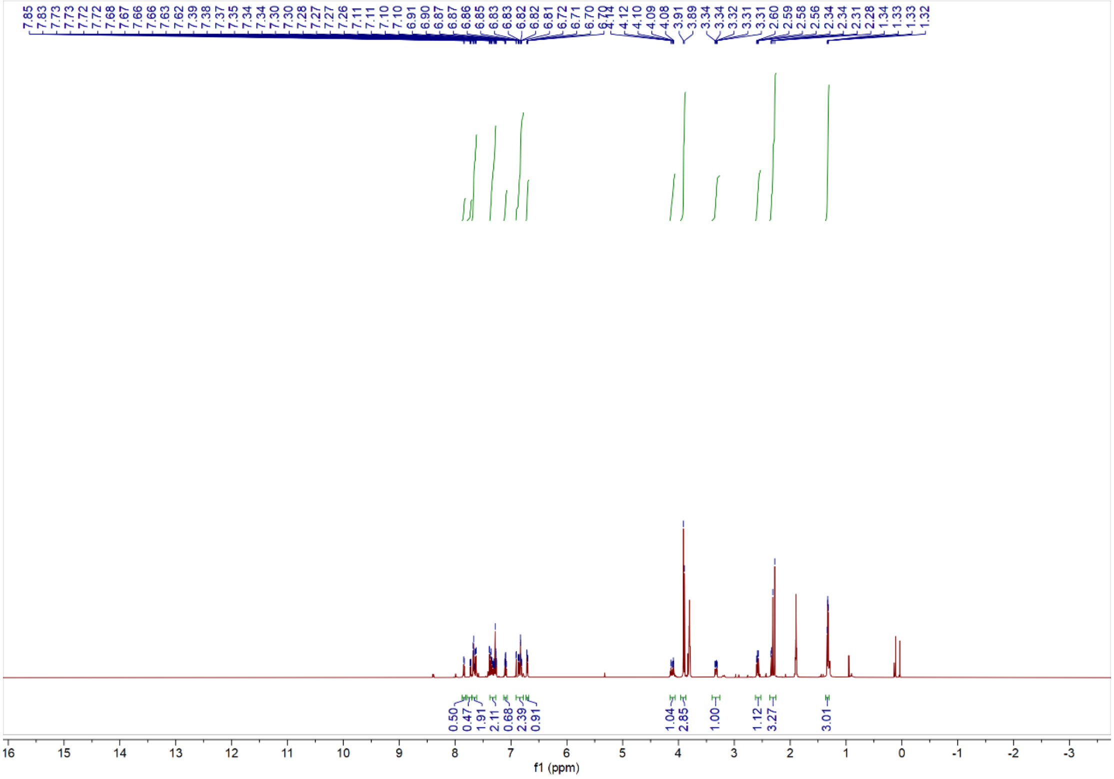

Figure S20 ^{1}H-NMR spectrum of **compound 15**

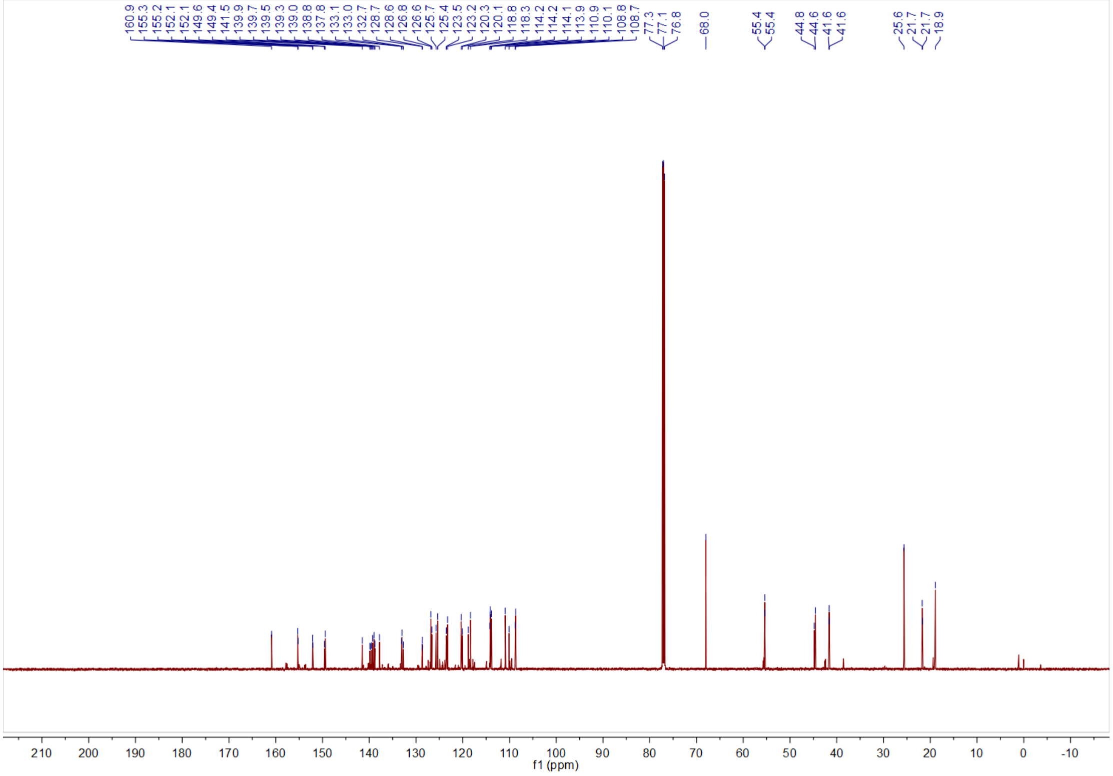

Figure S21 ^{13}C-NMR spectrum of compound 15

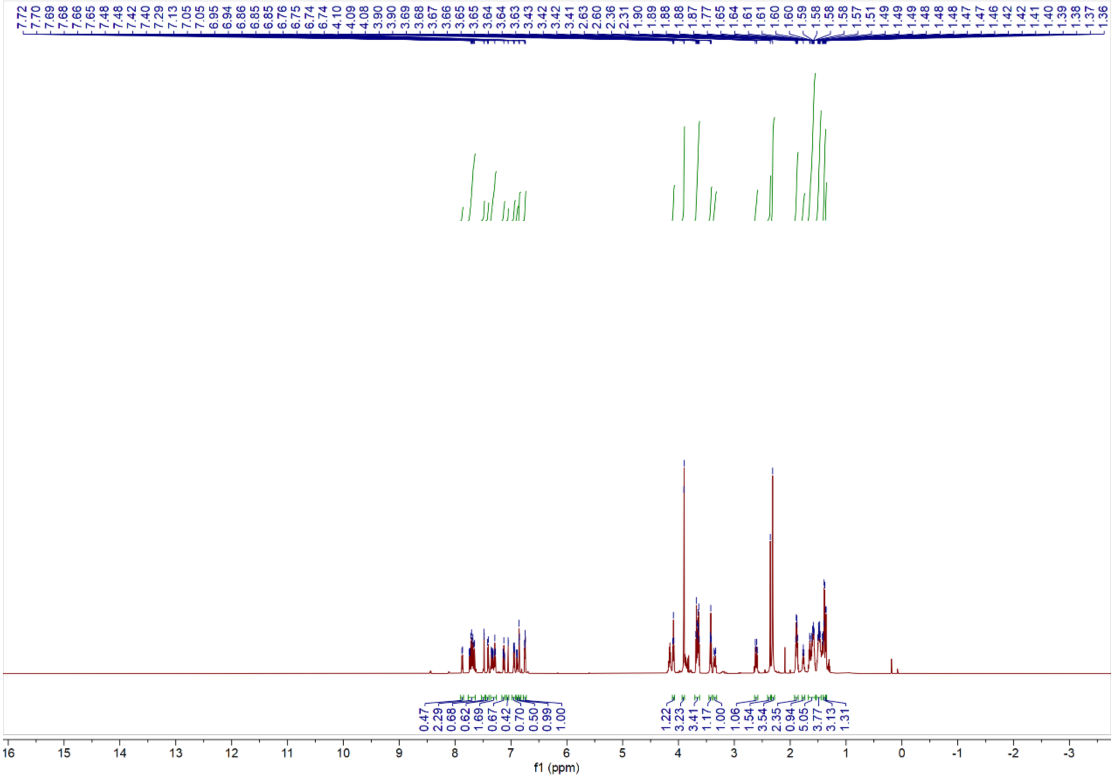

Figure S22 ^{1}H-NMR spectrum of **compound 16**

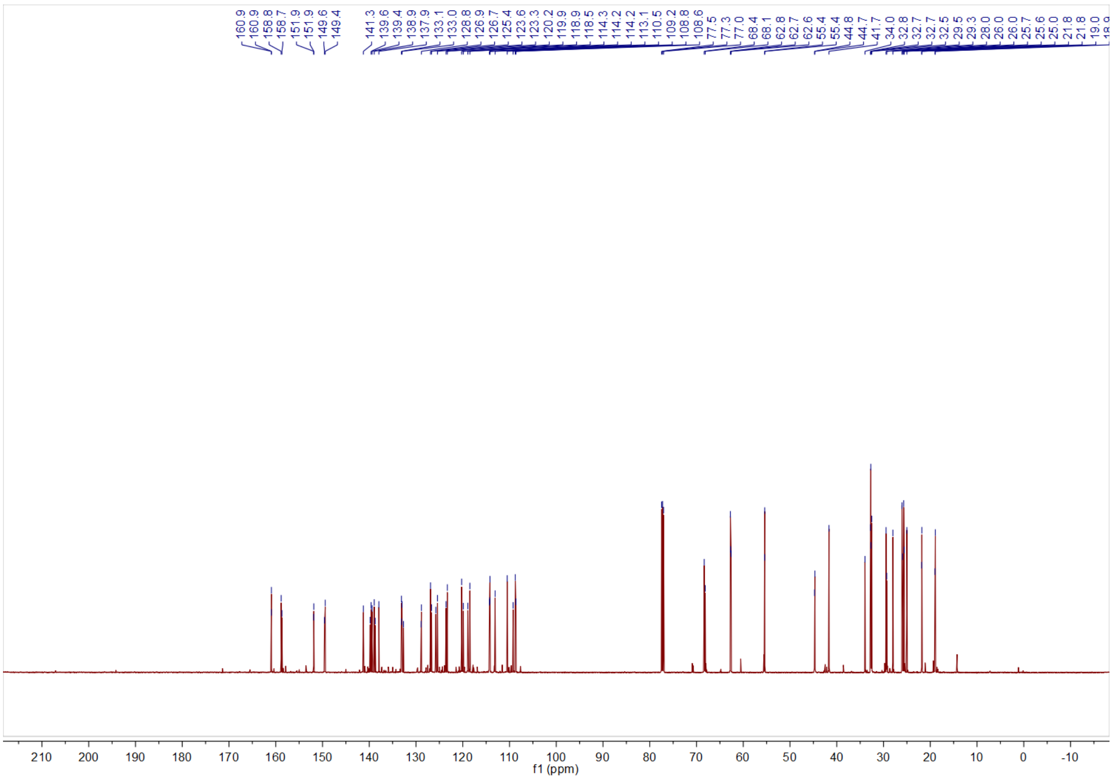

Figure S23 ^{13}C-NMR spectrum of compound 16

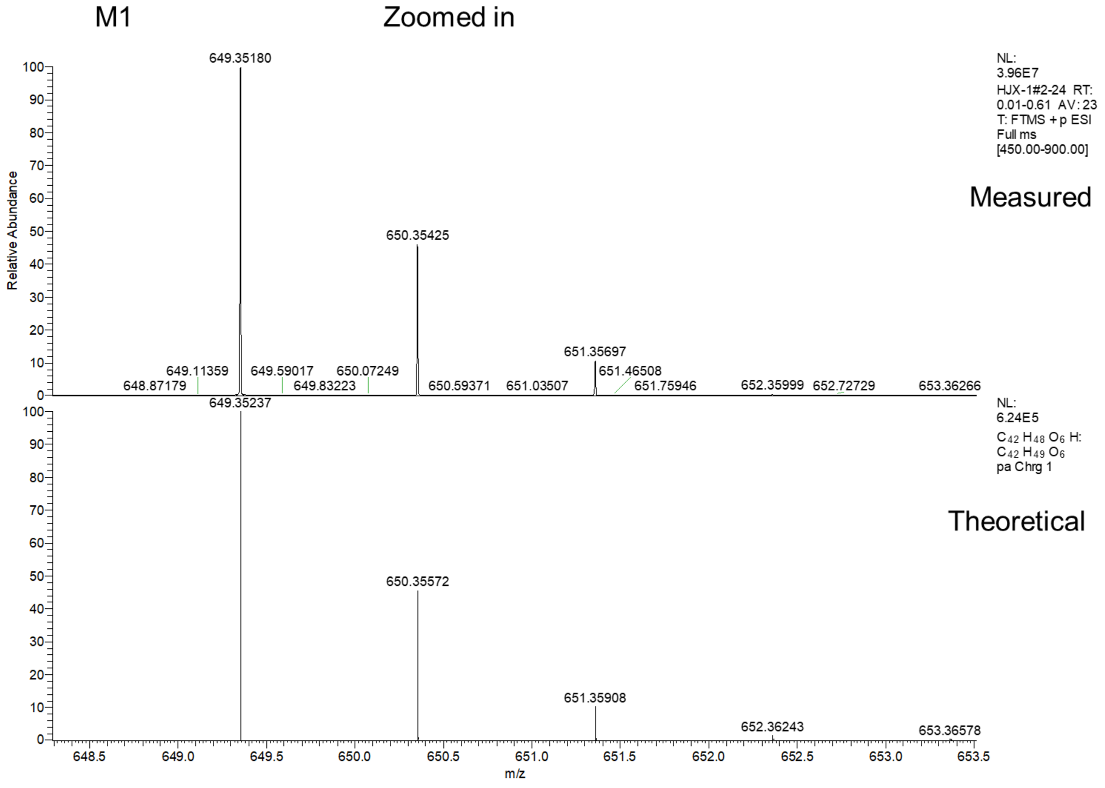

Figure S24 Mass spectrum of **M1**

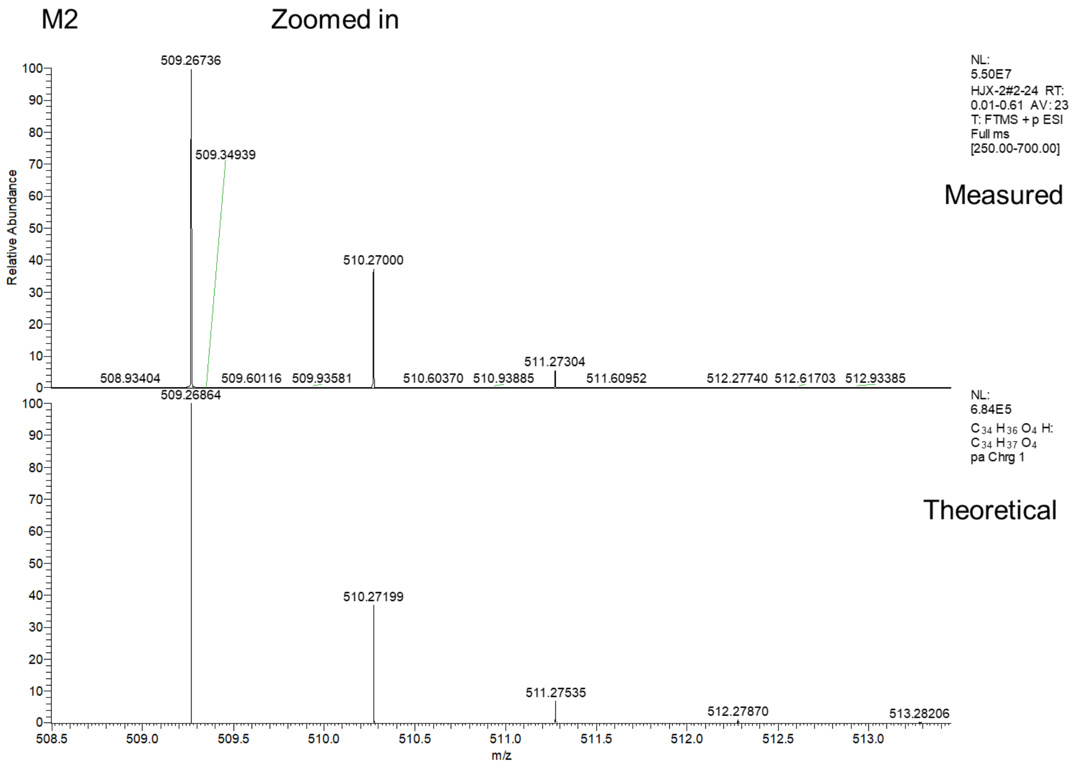

Figure S25 Mass spectrum of **M2**

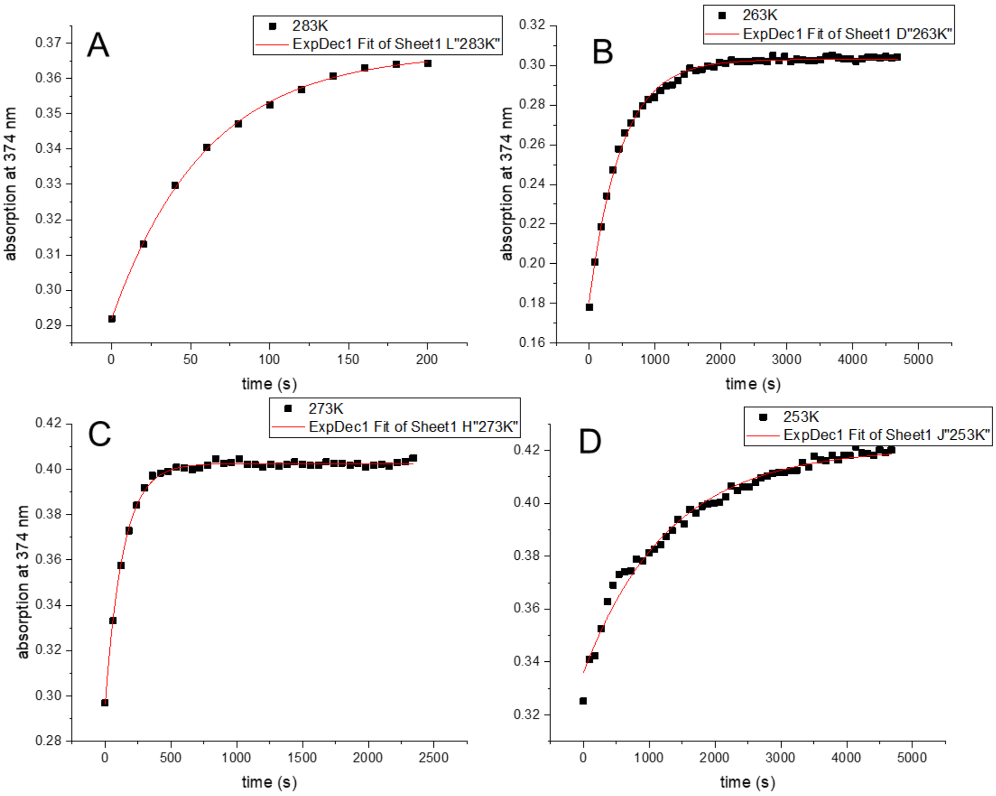

Figure S25 Change of UV absorption of **M1** at 374 nm from unstable-*Z* to stable Z isomer at (A) 283 K, (B) 273 K, (C) 263 K and (D) 253 K.

**Delta G**	7.93E+01 kJ/mol	**Delta G**	18.96 kcal/mol
**DH=**	61.14 kJ/mol	**DH=**	14.61 kcal/mol
**DS=**	-62.05 J/K.mol	**DS=**	-14.83 cal/K.mol

**DG**	79.33 kJ/mol	**DG**	18.96 kcal/mol
**DH**	61.14 kJ/mol	**DH**	14.61 kcal/mol
**DS**	-62.05 J/K.mol	**DS**	-14.83 cal/K.mol

**halflife**	15.5 sec
	0.3 min
	0.00 hours

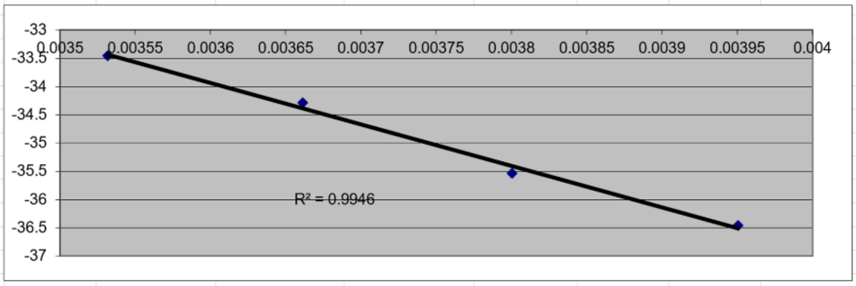

Figure S26 Eyring plot for the thermal helix inversion of unstable-*Z*-isomer to stable-*Z*-isomer. The linear fitting of ln (kh/k_0T) by 1/T using Erying equation $\ln\frac{k}{T} = \frac{-\Delta H^\ddagger}{R}\cdot\frac{1}{T} + \ln\frac{k_B}{h} + \frac{\Delta S^\ddagger}{R}$. The rate constants of the first-order decay k were obtained from

equation $A/A_0 = e^{-kt}$, at 283 K, 273 K, 263 K and 253 K. The half-lives ($t_{1/2}$) was calculated to be 12.9 s, respectively, for the unstable-*E* and unstable-*Z* isomers at room temperature.

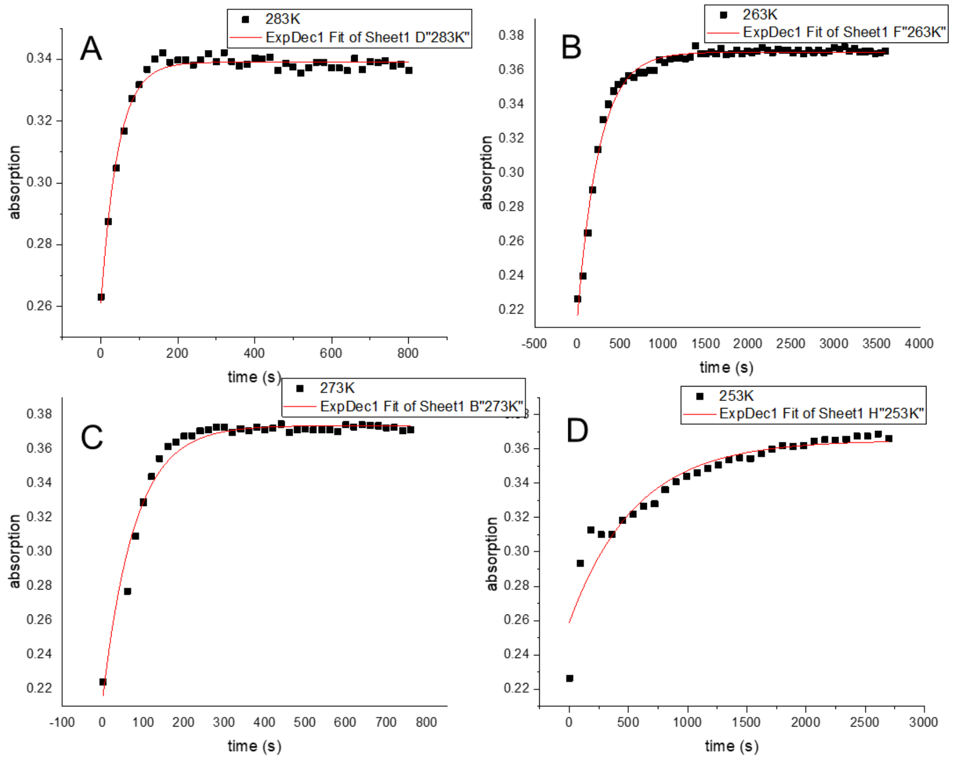

Figure S27 Change of UV absorption of **M1** at 374 nm from unstable-*E* to stable *E* isomer at (A) 283 K, (B) 273 K, (C) 263 K and (D) 253 K.

**Delta G**	7.89E+01 kJ/mol	**Delta G**	18.85 kcal/mol
**DH=**	51.98 kJ/mol	**DH=**	12.42 kcal/mol
**DS=**	-91.75 J/K.mol	**DS=**	-21.93 cal/K.mol

**DG**	78.87 kJ/mol	**DG**	18.85 kcal/mol
**DH**	51.98 kJ/mol	**DH**	12.42 kcal/mol
**DS**	-91.75 J/K.mol	**DS**	-21.93 cal/K.mol

**halflife**	12.9 sec
	0.2 min
	0.00 hours

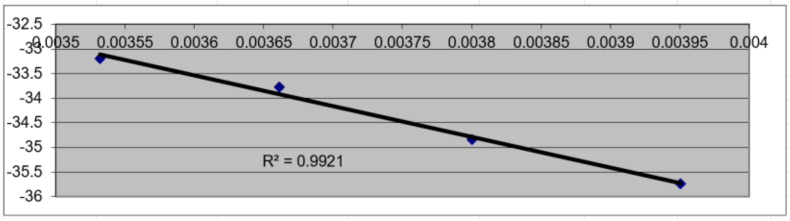

Figure S28 Eyring plot for the thermal helix inversion of unstable-*E*-isomer to stable-

E-isomer. The linear fitting of ln (kh/k_0T) by 1/T using Erying equation $\ln\frac{k}{T} = \frac{-\Delta H^{\ddagger}}{R}\cdot\frac{1}{T} + \ln\frac{k_B}{h} + \frac{\Delta S^{\ddagger}}{R}$. The rate constants of the first-order decay k were obtained from equation $A/A_o = e^{-kt}$, at 283 K, 273 K, 263 K and 253 K. The half-lives ($t_{1/2}$) was calculated to be 15.5 s at room temperature.

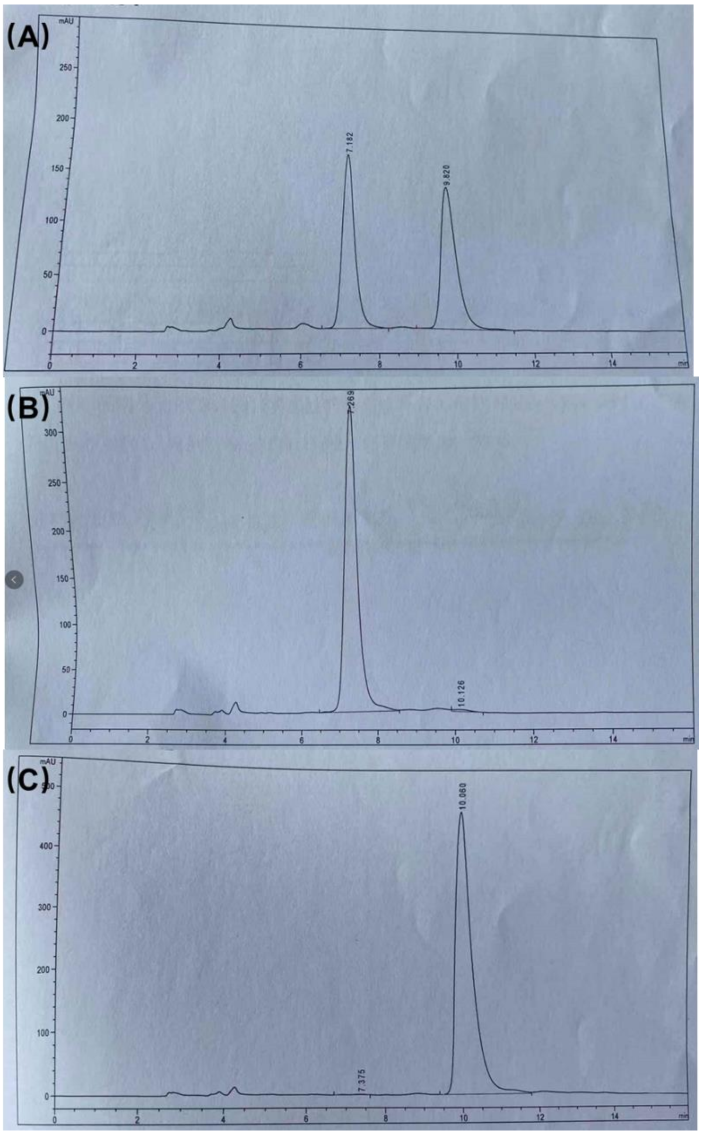

Figure S29 (A) HPLC spectra of racemic **M1** with HPLC. (B) HPLC spectra of (*R*)-**M1.** (C) HPLC spectra of (*S*)-**M1**. The separation and characterization were performed by HPLC using chiral stationary phase (Chiralpak AD, heptane : isopropanol 90 : 10) with a flow rate of 1.0 mL/min. We compared the CD spectra with the reported CD spectra of a motor with similar structure and assign the *R* and *S* enantiomers.[1]

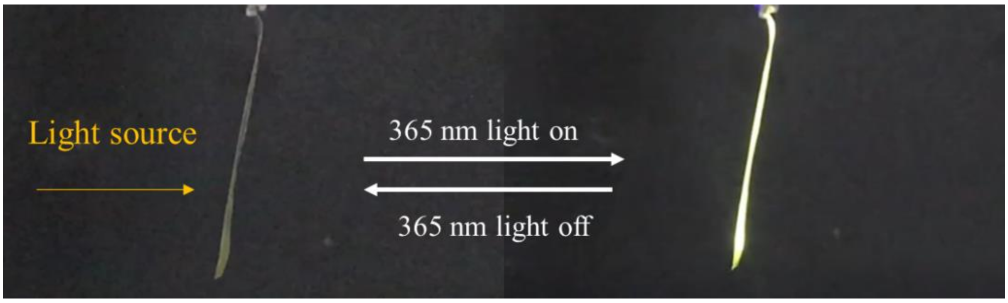

Figure S30 LC film prepared with **M2**. The mixture was aligned in a splayed cell with a thickness of 25 μm and cured by blue light. The cut ribbon showed no obvious motion upon UV irradiation. The ribbon was cut along the alignment.

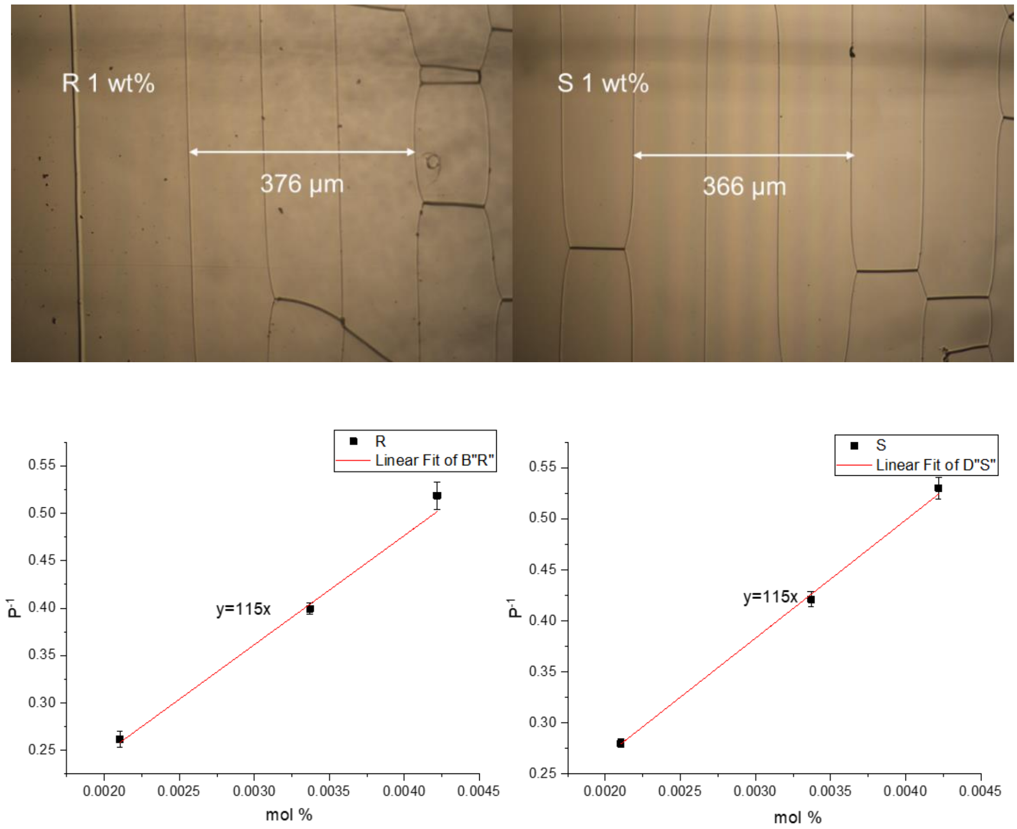

Figure S31 HTP measurement of **(*R*)-M1** and **(*S*)-M1** in E7.

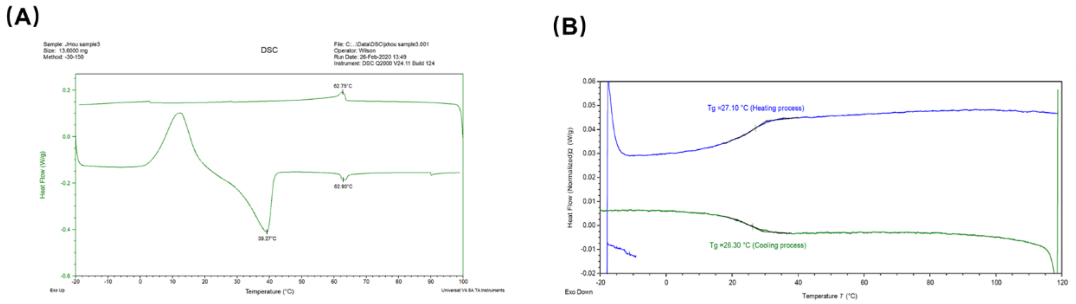

Figure S32 Differential Scanning Calorimetry spectra of LC mixture (3 wt% racemic **M1**, 18 wt% RM23, 31 wt% RM82, 46 wt% RM 105 and 2 wt% IRG 819) (A) before polymerization; (B) after polymerization.

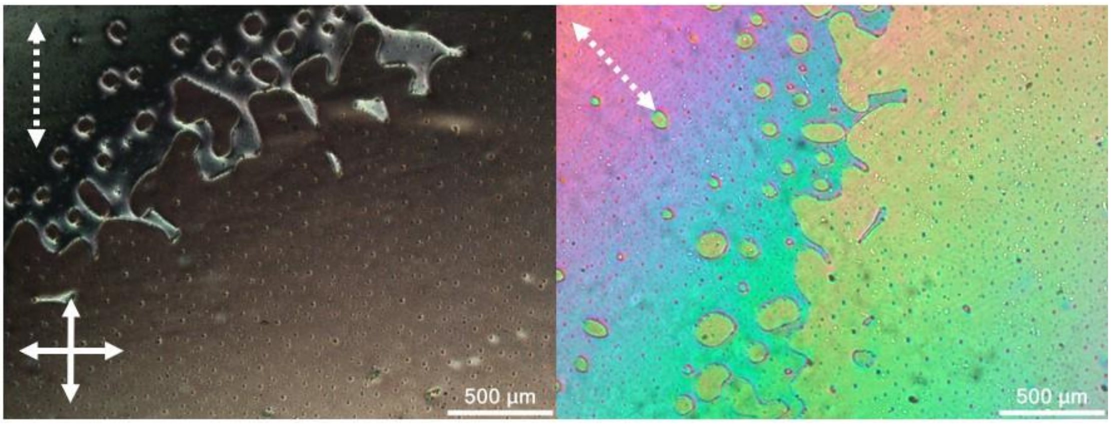

Figure S33 POM images of LC film (3 wt% racemic **M1**, 18 wt% RM23, 31 wt% RM82, 46 wt% RM 105 and 2 wt% IRG 819). The white cross represents the direction of the polarizers and the dash array represents the aligned direction of the LCN film at 90° and 45°.

Control Experiments:

In the control experiment, two commercially available chiral dopant R811 and S811 were mixed with azobenzene. Then 1 wt% R/S 811 and 1wt % A6MA were mixed together with the LC monomer mixture (18 wt% RM 23, 32 wt% RM 82, 46 wt% RM 105 and 2 wt% IRG 819) at 80 °C. The film was prepared following the same procedure as that of the motor film. The control film was cut into ribbons with a length of 3 cm and width with 5 mm. Irradiation experiment were performed in the same condition as the LC film with motor.

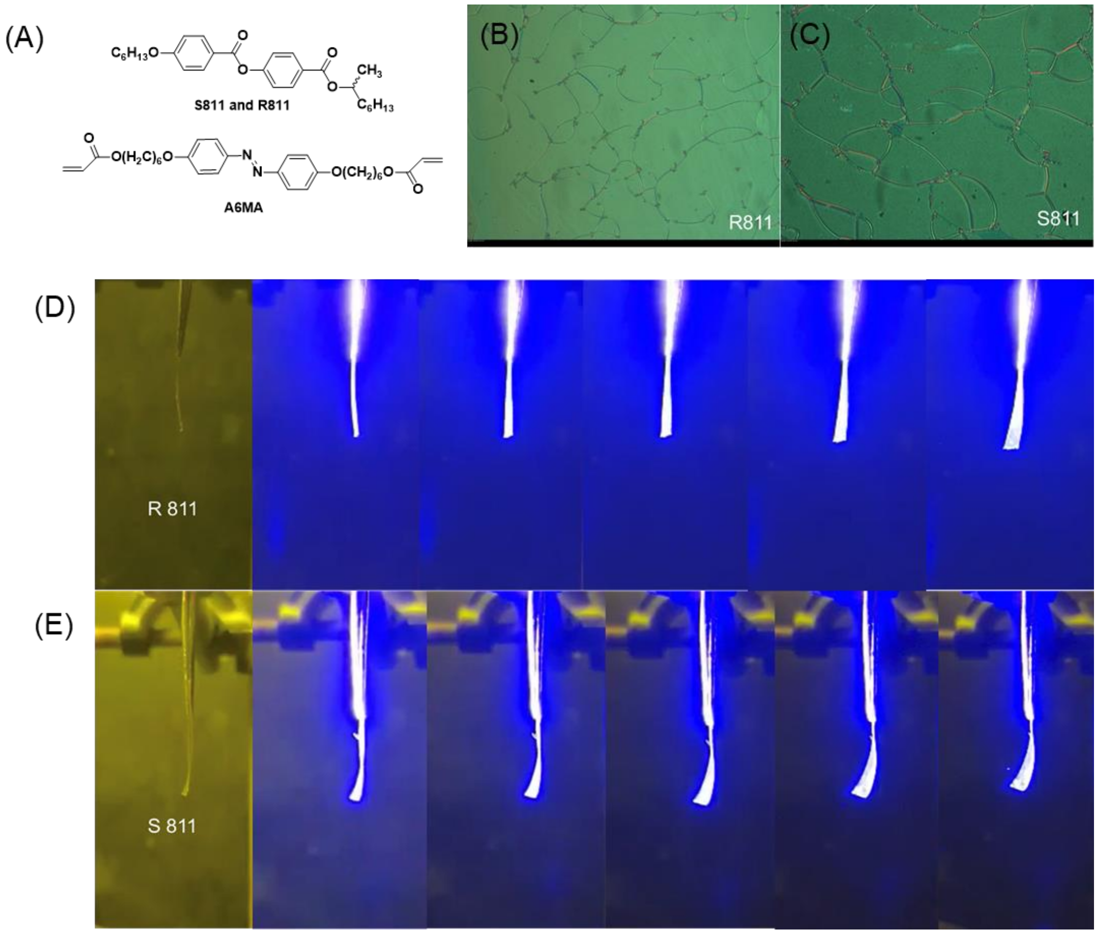

Figure S34 (A) Chemical structure of S/R 811 and A6MA (B) POM image of LC film with 1 wt% R811 (C) POM image of LC film with 1 wt% S811. (D) LC ribbons with R811 showed left-handed twisted motion upon UV irradiation. (E) LC ribbons with S811 showed right-handed twisted motion upon UV irradiation. All the ribbons were cut along the rubbing direction.

Reference:

[1] Gábor London, Kuang-Yen Chen, Gregory T. Carroll and Ben L. Feringa, Towards Dynamic Control of Wettability by Using Functionalized Altitudinal Molecular Motors on Solid Surfaces. *Chem. Eur. J.* **2013**, *19*, 10690-10697
